# Initiation of Genome Instability and Preneoplastic Processes through Loss of Fhit Expression

**DOI:** 10.1371/journal.pgen.1003077

**Published:** 2012-11-29

**Authors:** Joshua C. Saldivar, Satoshi Miuma, Jessica Bene, Seyed Ali Hosseini, Hidetaka Shibata, Jin Sun, Linda J. Wheeler, Christopher K. Mathews, Kay Huebner

**Affiliations:** 1 Biomedical Sciences Graduate Program, Ohio State University, Columbus, Ohio, United States of America; 2 Department of Molecular Virology, Immunology, and Medical Genetics, Ohio State University Comprehensive Cancer Center, Columbus, Ohio, United States of America; 3 Department of Biochemistry and Biophysics, Oregon State University, Corvallis, Oregon, United States of America; University of Washington, United States of America

## Abstract

Genomic instability drives tumorigenesis, but how it is initiated in sporadic neoplasias is unknown. In early preneoplasias, alterations at chromosome fragile sites arise due to DNA replication stress. A frequent, perhaps earliest, genetic alteration in preneoplasias is deletion within the fragile FRA3B/*FHIT* locus, leading to loss of Fhit protein expression. Because common chromosome fragile sites are exquisitely sensitive to replication stress, it has been proposed that their clonal alterations in cancer cells are due to stress sensitivity rather than to a selective advantage imparted by loss of expression of fragile gene products. Here, we show in normal, transformed, and cancer-derived cell lines that Fhit-depletion causes replication stress-induced DNA double-strand breaks. Using DNA combing, we observed a defect in replication fork progression in Fhit-deficient cells that stemmed primarily from fork stalling and collapse. The likely mechanism for the role of Fhit in replication fork progression is through regulation of Thymidine kinase 1 expression and thymidine triphosphate pool levels; notably, restoration of nucleotide balance rescued DNA replication defects and suppressed DNA breakage in Fhit-deficient cells. Depletion of Fhit did not activate the DNA damage response nor cause cell cycle arrest, allowing continued cell proliferation and ongoing chromosomal instability. This finding was in accord with *in vivo* studies, as Fhit knockout mouse tissue showed no evidence of cell cycle arrest or senescence yet exhibited numerous somatic DNA copy number aberrations at replication stress-sensitive loci. Furthermore, cells established from *Fhit* knockout tissue showed rapid immortalization and selection of DNA deletions and amplifications, including amplification of the *Mdm2* gene, suggesting that Fhit loss-induced genome instability facilitates transformation. We propose that loss of Fhit expression in precancerous lesions is the first step in the initiation of genomic instability, linking alterations at common fragile sites to the origin of genome instability.

## Author Summary

Normal cells have robust mechanisms to maintain the proper sequence of their DNA; in cancer cells these mechanisms are compromised, resulting in complex changes in the DNA of tumors. How this genome instability begins has not been defined, except in cases of familial cancers, which often have mutations in genes called “caretaker” genes, necessary to preserve DNA stability. We have defined a mechanism for genome instability in non-familial tumors that occur sporadically in the population. Certain fragile regions of our DNA are more difficult to duplicate during cell division and are prone to breakage. A fragile region, FRA3B, lies within the *FHIT* gene, and deletions within FRA3B are common in precancer cells, causing loss of Fhit protein expression. We find that loss of Fhit protein causes defective DNA replication, leading to further DNA breaks. Cells that continue DNA replication in the absence of Fhit develop numerous chromosomal aberrations. Importantly, cells established from tissues of mice that are missing Fhit undergo selection for increasing DNA alterations that can promote immortality, a cancer cell hallmark. Thus, loss of Fhit expression in precancer cells is the first step in the initiation of genomic instability and facilitates cancer development.

## Introduction

Genomic instability drives tumorigenesis by expediting the acquisition of mutations that provide for selective clonal expansion and escape of normal cellular restraints [1]. Expressions of genome instability include chromosomal instability, microsatellite instability, and instabilities typified by an increased frequency of point mutations. Chromosomal instability is the most commonly observed form of genome instability, occurs in the majority of sporadic cancers and includes structural chromosome aberration (translocations, inversions, deletions and duplications) or numerical abnormality (aneuploidy, triploidy, tetraploidy.) [2]. Because of its occurrence in most cancers, the molecular events causing chromosome and genome instability have been the subject of intense investigation. Chromosome and genome instability terminology is used interchangeably in this study to refer to chromosome structural and numerical abnormalities.

In several familial cancer syndromes, genome instability develops due to inherited mutations in the “DNA caretaker” genes essential for DNA repair or the DNA damage response [3]. However, in sporadic cancers the known DNA caretaker genes are rarely mutated before the rise of genome instability [2]. It has been proposed that in early stages of sporadic tumorigenesis, activated oncogenes induce replication stress through deregulation of cell cycle progression, causing chromosomal instability, first at common fragile sites, and later throughout the genome [4], [5]. This proposal was corroborated by a report that expression of activated oncogenes *in vitro* results in nucleotide pool levels inadequate to support normal DNA replication, due to premature S phase entry [6]. Remarkably, exogenously supplied nucleosides suppressed oncogenesis in the model systems studied.

While oncogene activation can induce replication stress *in vitro* and in mouse models, it also activates DNA damage response checkpoints and causes cellular senescence, forming a barrier to cancer progression [7], [8]. Without inactivating mutations in DNA damage response genes or experimental manipulation of cell cycle checkpoints, transformation does not occur. This suggests that genome instability or mutational diversity in genetically un-manipulated models occurs prior to oncogene activation. Moreover, recent studies have detected the presence of clonal somatic mosaicism in a small fraction of healthy individuals. These chromosomal anomalies are more prevalent in older individuals, and precede oncogenesis [9], [10], in accord with the idea that genome instability occurs prior to oncogene activation. It has also been argued that since cancers with microsatellite instability, but not chromosomal instability, similarly express the activated oncogenes proposed to induce chromosomal instability, such oncogenes do not actually cause chromosome alterations [11]. Finally, genomic alterations are observed in human precancerous lesions, yet there are few reports of activated oncogenes in such lesions. Thus, oncogene-induced DNA damage contributes to the progression of genome instability in sporadic cancers, but is unlikely to initiate it.

Deletions at common fragile site FRA3B do occur in preneoplasias and may be the most frequent and earliest alterations. FRA3B overlaps the *FHIT* gene, and FRA3B fragility often results in deletions of *FHIT* exons and loss of Fhit expression in precancer and cancer cells [12]. Paradoxically, examination of cells that have lost the *FHIT* gene product has revealed that Fhit protein has functional roles in response to DNA damage [13]: 1) kidney epithelial cells established from *Fhit* ^−/−^ mice exhibited >2-fold increased chromosome breaks at fragile sites *vs* corresponding *Fhit* ^+/+^ kidney cells [14]; 2) the frequency of mutations following replicative or oxidative stress in Fhit-deficient transformed and cancer cells was 2 to 5-fold greater than in Fhit-expressing cells [15], [16]. Despite these findings and strong evidence that Fhit exerts tumor suppressor activity [17], [18], it has been argued that deletions within the *FHIT* locus in transformed cells are passenger alterations rather than cancer-driving mutations [19]. In this study we have further examined the role of Fhit loss in development of DNA damage and observed that absence of Fhit causes genome instability without activating the DNA damage response and senescence barrier. Our findings support a model for the initiation of genome instability in early stages of neoplasia through *FHIT*/FRA3B alterations and subsequent loss of Fhit function.

## Results

### Fhit-deficient cells exhibit spontaneous DNA breaks

**Figure 1:**
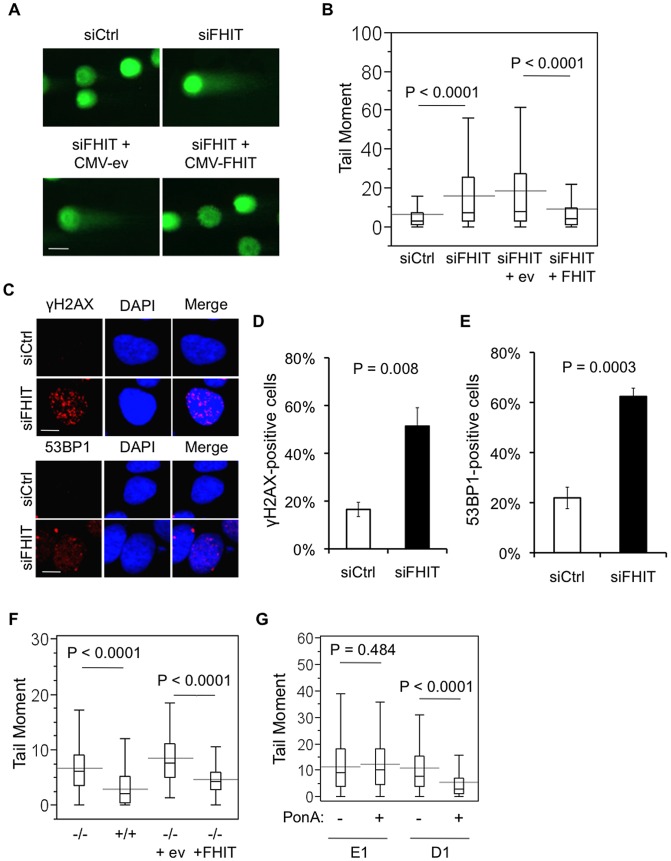
Fhit-deficient cells exhibit spontaneous DNA breaks. (A) Neutral comet assays of HEK293 cells 2 days after transfections with siRNAs and pRcCMV-*FHIT*-flag or pRcCMV-empty-flag plasmids. Representative nuclei are shown; bars, 20 µm. (B) Box plots of Tail moments include data (siCtrl, n = 183; si*FHIT*, n = 142; si*FHIT*+CMV-ev, n = 135; si*FHIT*+CMV-Fhit, n = 132) from 3 separate experiments. Statistical significance was determined using the Kruskal-Wallis rank sum test. (C) Indirect immunofluorescence of γH2AX and 53BP1, 2 days after Fhit knockdown. Representative nuclei are shown; bars, 10 µm. (D and E) Quantification of γH2AX-positive cells (D) and 53BP1-positive cells (E). Bar graphs indicate the means, and error bars represent the standard deviations. Data were collected from 3 independent experiments. Statistical significance was assessed using a 2-sided Student's T-test. (F) Neutral comet assays of *Fhit* ^+/+^ or *Fhit* ^−/−^ mouse kidney cells 48 h after transfection with pRcCMV-*FHIT*-flag or pRcCMV-empty-flag plasmids. Box plots of Tail moments are shown. Statistical significance was determined using the Mann-Whitney rank sum test. (G) Neutral comet assays of Fhit-deficient H1299 lung carcinomas cells, with or without induction of Fhit expression. Comet assays were performed 48 h after ponasterone A-induction of Fhit expression. Box plots of Tail moments are shown. Statistical significance was determined using the Mann-Whitney rank sum test.

To define the role of Fhit in promoting genome stability, we began by assessing spontaneous DNA damage in HEK293 embryonic kidney cells transfected with siRNAs targeting *FHIT* (Figure S1A). DNA damage was measured by neutral comet assay, or single cell gel electrophoresis assay, a method routinely used to detect DNA double-strand breaks (DSBs). The assay is based on the principle that fragmented DNA migrates faster than un-fragmented DNA through agarose gel in an electric field. Undamaged DNA remains in the nucleoid and is seen as the “comet head”, while damaged DNA migrates through the gel and forms the “comet tail” (Figure 1A). We used the tail moment, the product of tail length and % of total DNA in the tail, to score DSB levels in individual cells. Two days following Fhit knockdown, we observed a significant increase in the mean tail moment in Fhit-deficient cells *vs* si-control cells, indicating that decreased Fhit expression resulted in spontaneous DSBs. Co-transfection with *FHIT* siRNAs and a *FHIT* expression plasmid suppressed DSB formation, confirming that Fhit-depletion caused the DSBs (Figure 1B). As independent confirmation of DSBs following Fhit knockdown, we assessed numbers of nuclear γH2AX and 53BP1 foci, markers of DNA breaks, by indirect immunofluorescence (Figure 1C). HEK293 cells transfected with *FHIT* siRNAs exhibited a ∼3-fold increase in the fraction of cells with γH2AX and 53BP1 foci (Figure 1D and 1E), confirming that loss of Fhit expression causes DSBs. To determine if Fhit prevents DSBs in normal cells, we compared tail moments in primary cells established from *Fhit* ^−/−^ mouse kidney *vs* cells from wild-type *Fhit* ^+/+^ kidney. The mean tail moment of *Fhit^−/−^* cells was ∼2-fold greater than that of *Fhit^+/+^* cells and exogenous Fhit expression in the *Fhit^−/−^* cells decreased the mean tail moment (Figure 1F). To determine if Fhit suppresses formation of DSBs in cancer cells, we used endogenous Fhit-negative H1299 lung adenocarcinoma cells carrying either an inducible *FHIT* cDNA expression plasmid (D1 clone) or the empty vector control (E1 clone) (Figure S1B). After induction of Fhit expression in D1 cells, the mean tail moment was significantly reduced (Figure 1G). Collectively, these results demonstrate the critical role for Fhit in suppressing spontaneous DSBs in transformed cells and cells derived from normal and cancerous tissue.

### Fhit prevents endogenous replication stress and replication fork stalling

**Figure 2:**
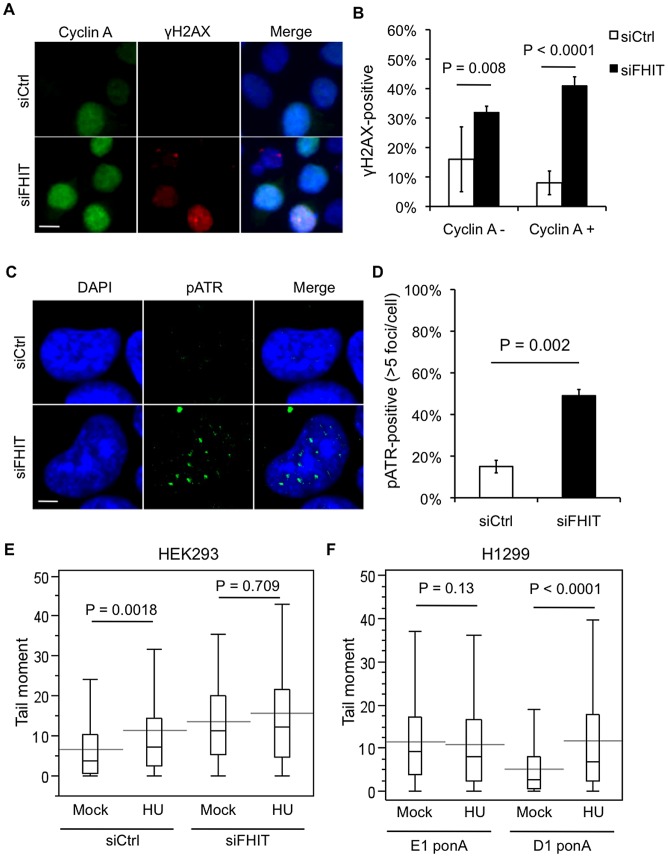
Loss of Fhit causes replication stress. (A) Cyclin A and γH2AX indirect immunofluorescence after Fhit knockdown. Representative images are shown; bars, 10 µm. (B) Data obtained in (A) were quantified from 3 independent experiments, and statistical significance was determined using a 2-sided T-test. Bar graphs represent the means, and error bars mark the standard deviations. (C) pATR immunofluorescence 2 days after Fhit knockdown in HEK293 cells. Representative images are shown; bars, 5 µm. (D) Quantification of cells positive for more than 5 pATR foci/cell from 3 independent experiments; statistical significance was determined using a 2-sided T-test. (E) Neutral comet assays in siRNA transfected HEK293 cells treated with 2 mM hydroxyurea for 4 h. Box plots show quantification of Tail moments. P-values were determined using the Mann-Whitney rank sum test. (F) Neutral comet assays in H1299 E1 and D1 cells with ponasterone A-induction treated with 2 mM hydroxyurea for 4 h. Box plots show quantification of Tail moments. P-values were determined using the Mann-Whitney rank sum test.

Since endogenous DSBs typically form due to DNA replication defects [20], we asked if Fhit-deficient cells exhibit increased replication stress. We co-immunostained Fhit-silenced HEK293 cells with antisera against γH2AX and Cyclin A, an S/G2 phase marker. Fhit-deficient cells exhibited a dramatic increase in γH2AX immunofluorescence staining in Cyclin A-positive cells (Figure 2A and 2B), suggesting that DNA breaks were caused by aberrant DNA replication. Similar results were obtained using H1299 cells, as induction of Fhit expression in D1 cells led to reduced numbers of γH2AX foci in Cyclin A-positive cells (Figure S2A and S2B). To determine if DNA damage occurred specifically at sites of replication, we immunostained for phospho-ATR (Ser428), a kinase that localizes to stalled replication forks and initiates the S phase checkpoint [21]. Fhit knockdown resulted in a ∼3-fold increase in the fraction of cells with phospho-ATR (Ser428) nuclear foci *vs* HEK293 cells treated with control siRNAs (Figure 2C and 2D). To verify that replication forks were more frequently damaged in Fhit-deficient cells, we pulse-labeled H1299 E1 and D1 cell replication forks with BrdU, and immunostained for γH2AX and BrdU. In these cells, induction of Fhit expression in D1 cells led to reduced γH2AX localization to BrdU-labeled replication foci (Figure S2C and S2D). We conclude that Fhit functions to decrease DNA damage arising from endogenous replication stress, a conclusion supported by the observation that inhibition of S phase by roscovitine treatment, a Cdk inhibitor, suppressed γH2AX staining in Fhit-deficient H1299 E1 cells (Figure S2E and S2F).

We reasoned that Fhit may either function upstream to prevent and minimize DNA replication stress or, alternatively may contribute to downstream replication fork maintenance and thereby prevent fork collapse and DSB formation. To distinguish between a role of Fhit upstream or downstream of replication stress, we treated cells with hydroxyurea for 4 h and measured comet tail moments. Hydroxyurea causes replication fork stalling through depletion of dNTPs by inhibition of ribonucleotide reductase; thus cells treated with hydroxyurea for more than 2 h accumulate inactivated replication forks and DSBs [22], [23]. If Fhit functions to support replication fork stability after replication stress, then hydroxyurea challenge should induce more DSBs in Fhit-deficient cells. However, hydroxyurea treated Fhit-silenced HEK293 cells and treated control cells exhibited similar levels of comet tail moments (Figure 2E). Similar results were obtained in H1299 D1 and E1 cells (Figure 2F). Overall, hydroxyurea treatment resulted in equivalent tail moments in Fhit-expressing and Fhit-deficient cells, suggesting that Fhit does not function downstream of replication stress.

**Figure 3:**
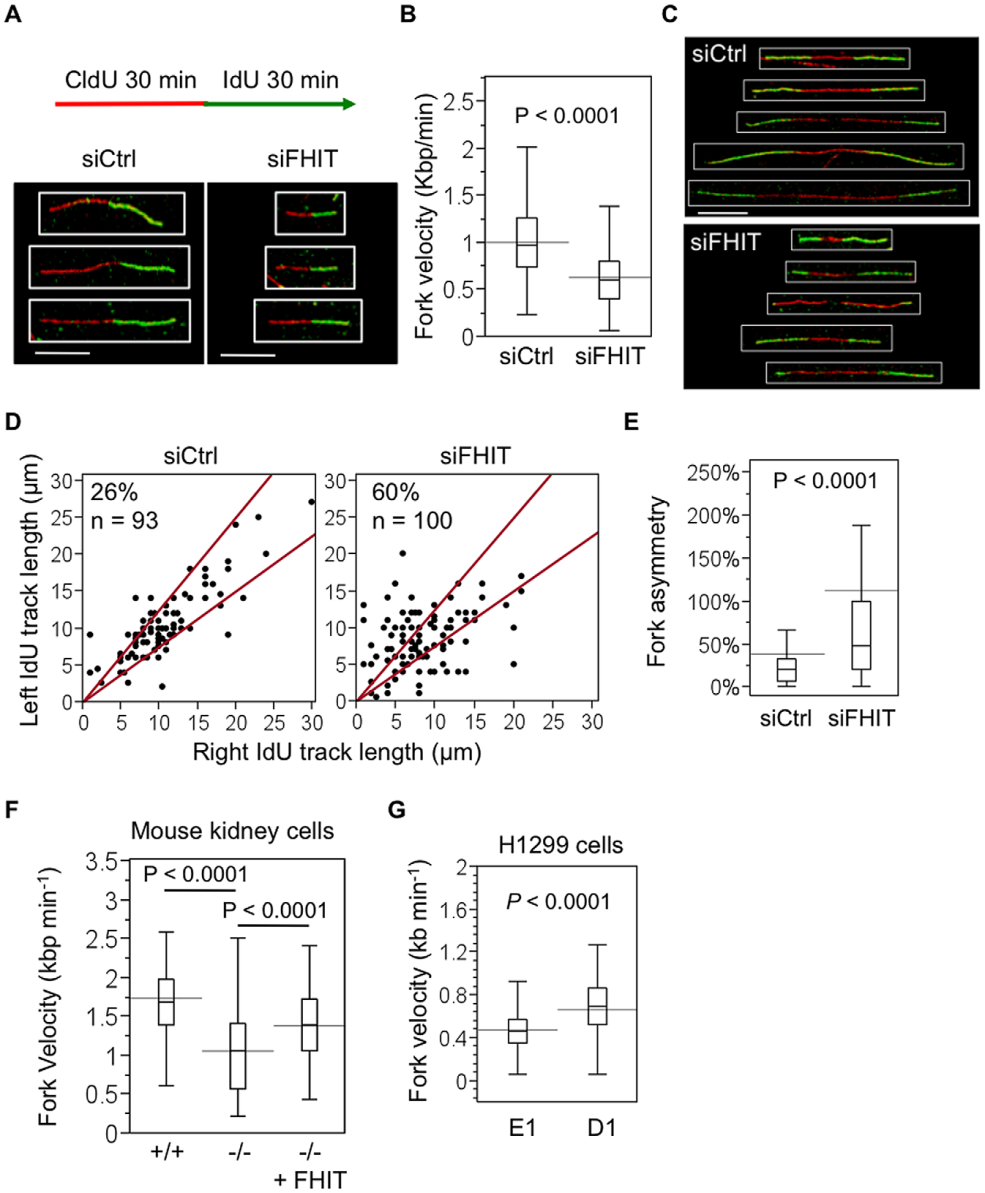
Loss of Fhit expression causes fork stalling. (A) siRNA transfected HEK293 cells were pulse-labeled with CldU for 30 min, washed and pulse-labeled with IdU for 30 min. Representative indirect immunofluorescence images of labeled fibers are shown; bars, 10 µm. (B) Quantification of fork velocity in HEK293 cells. Fork velocity was determined by measuring lengths (µm) of IdU-labeled fibers and converting to kbp using a conversion factor of 1 µm = 2.59 kbp. Bars extending through boxplots indicate mean velocity, and bars contained within boxplots indicate median velocity. Statistical significance was determined using a 2-sided Student's T-test (n = 238 for siCtrl; n = 320 for siFHIT). (C) Representative images of sister forks proceeding outward from a common origin in siRNA transfected HEK293 cells; bars, 10 µm. DNA fibers from siRNA transfected HEK293 cells were prepared as in (A). (D) Scatter plots of distances traveled by left and right sister forks during pulse-labeling with IdU. The central area marked by red lines represents sister forks with less than 25% length difference. The percentages of asymmetric sister forks are indicated at the upper left region of plots. (E) Fork asymmetry is calculated as the ratio of the longest IdU tract to the shortest for each pair of sister forks. P-value was determined using the Mann-Whitney rank sum test. (F) DNA fiber analysis of fork velocity in *Fhit* ^+/+^, *Fhit* ^−/−^ and *Fhit* ^−/−^ pRcCMV-*FHIT*-flag plasmid transfected mouse kidney cells. Quantification and statistical analysis was performed as described in (B). (G) DNA fiber analysis of fork velocity in H1299 E1 and D1 cells 48 h after ponasterone A treatment. Quantification and statistical analysis was performed as described in (B).

Next we investigated the possibility that Fhit supports normal DNA replication, such that silencing Fhit causes replication stress, through analysis of DNA replication dynamics at the single-molecule level by DNA combing [24]. DNA fibers from HEK293 cells pulsed sequentially 30 min each with the nucleotide analogs, 5-chlorodeoxyuridine (CldU) and 5-iododeoxyuridine (IdU), were spread on glass slides. Replicating DNA incorporates CldU and then IdU during the sequential pulses, and is detected by immunofluorescence (Figure 3A). Labeled DNA fibers were consistently shorter in cells transfected with *FHIT* siRNAs *vs* control siRNAs. Using a conversion factor of 1 µm = 2.59 kilobase pairs (kbp) [24], average fork velocities of 1.05 kbp/min were estimated for control and 0.61 kbp/min for Fhit-silenced cells (Figure 3B). The results illustrate the role of Fhit in sustaining normal DNA replication. We also assessed the symmetry of sister replication forks proceeding outward from common replication origins that fired during the CldU pulse (Figure 3C). Because DNA synthesis is coupled at sister replication forks, asymmetrical DNA synthesis is thought to represent stalling or collapse of one of the forks [25]. Control cells exhibited mostly symmetrical sister forks, with length of IdU-labeled tracts on either side of the replication origin nearly equal. In contrast, Fhit knockdown increased the frequency of asymmetrical sister forks proceeding outward from a common origin (Figure 3C–3E), suggesting that Fhit loss results in increased fork stalling and collapse. We also assessed replication dynamics in mouse kidney cells and H1299 cancer cells; replication defects were also observed in *Fhit* ^−/−^ mouse kidney cells and Fhit-deficient H1299 cells (Figure 3F and 3G). Overall, the results suggest that Fhit does not participate in the response to fork stress, but rather, in unperturbed conditions Fhit promotes normal DNA replication progression.

### Fhit modulates the supply of dTTP

**Figure 4:**
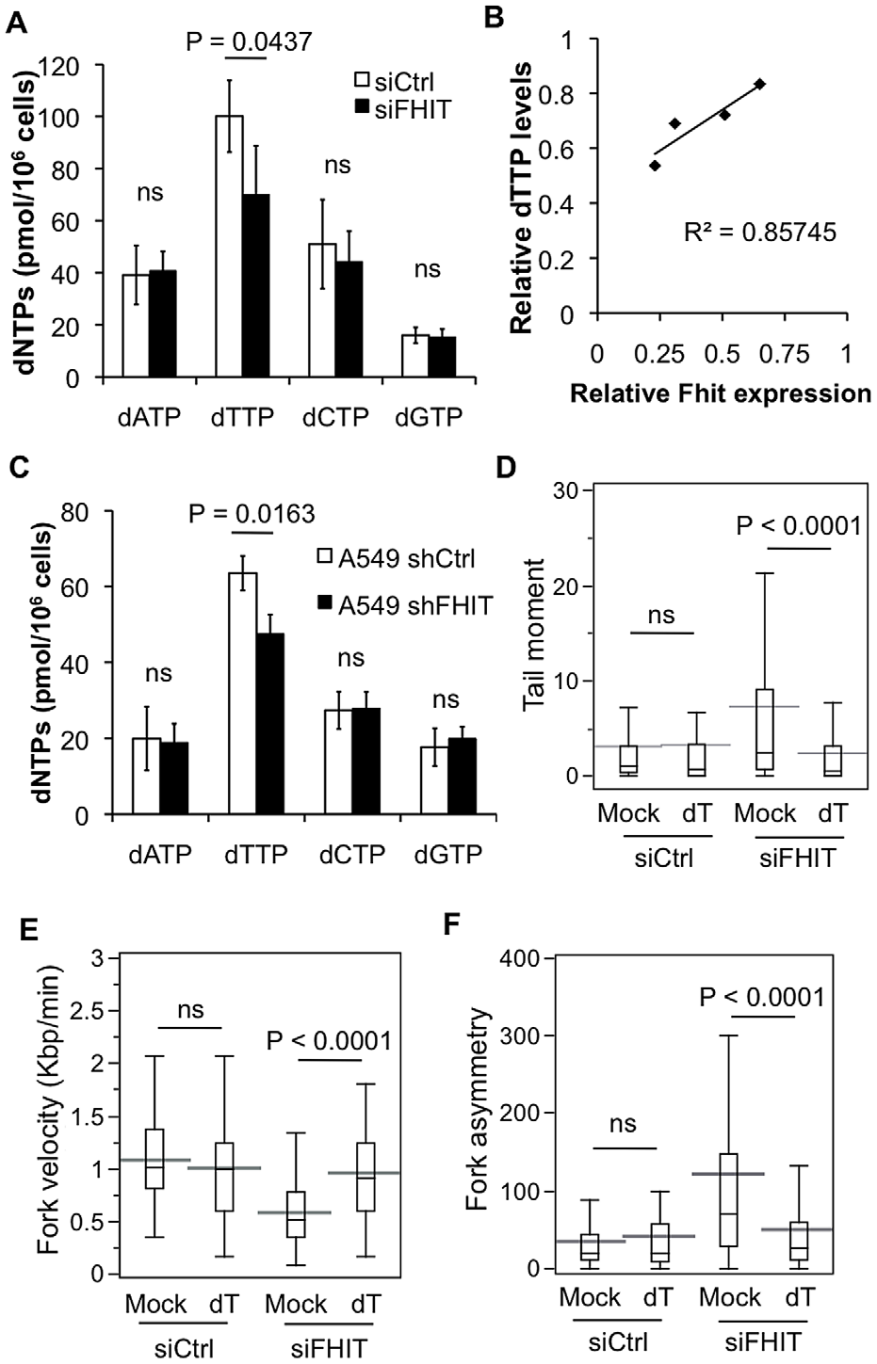
Fhit modulates dTTP pools to prevent DNA breaks. (A) Deoxyribonucleotide triphosphate (dNTP) levels in HEK293 cells 72 h after Fhit knockdown. Bar graphs represent means of 4 independent experiments, and error bars denote the standard deviations. The P-values were determined using a 2-sided T test; ns = not significant. (B) Correlation of relative Fhit expression and relative dTTP levels. siRNA transfected HEK293 cells were split into matching pairs, one for dNTP analysis and the other for western blot analysis of Fhit knockdown. Relative Fhit expression in Fhit knockdown cells compared to control cells was determined by densitometry and normalized to GAPDH expression. Relative dTTP levels were defined as dTTP concentration in siFHIT cells/dTTP concentration in siCtrl cells. (C) dNTP measurements in A549 cells with 7–9 week stable Fhit knockdown. Bar graphs represent means of 7 independent experiments, and error bars denote the standard deviation. P-values were calculated as in (A). (D) Box plots of Tail moments measured from neutral comet assays of HEK293 cells with Fhit knockdown, untreated or supplemented daily with 10 µM thymidine for 48 h (siCtrl mock, n = 242; siCtrl+thymidine, n = 156; siFHIT mock, n = 193; siFHIT+thymidine, n = 115). Statistical significance was determined using the Kruskal-Wallis rank sum test. (E) DNA fiber analysis of fork velocity in siRNA transfected HEK293 cells supplemented daily with 10 µM thymidine for 48 h. Statistical significance was determined using a 2-sided Student's T-test (siCtrl mock, n = 136; siCtrl+thymidine, n = 152; siFHIT mock, n = 155; siFHIT+thymidine, n = 153). (F) DNA fiber analysis of sister fork asymmetry in siRNA transfected HEK293 cells supplemented daily with 10 µM thymidine for 48 h. Fork asymmetry and P-values were determined as in Figure 3E (siCtrl mock, n = 87; siCtrl+thymidine, n = 86; siFHIT mock, n = 96; siFHIT+thymidine, n = 93). dT = thymidine 10 µM; ns = not significantly different.

To explore mechanisms involved in Fhit support of DNA replication progression, we considered the observation that depletion of dNTP pools by hydroxyurea exposure equalized DNA damage in Fhit-positive and deficient cells (Figure 2E and 2F). It is known that imbalance in dNTPs is mutagenic and produces chromosomal abnormalities [26], and in certain models, reduced dNTP pools aid transformation [6]. To determine if such imbalances occur spontaneously following knockdown of Fhit expression, we measured dNTP pools using the enzymatic assay developed by Sherman and Fyfe [27]. Within 72 h, Fhit knockdown in HEK293 cells caused ∼30% reduction in the dTTP level compared to control cells, with other dNTPs unaffected (Figure 4A). In these experiments Fhit knockdown relative to control cells ranged from 35–75%; to account for differences in knockdown efficiency, we plotted the relative Fhit expression *vs* the relative dTTP levels, revealing that cells with greater knockdown of Fhit expression had lower levels of dTTP, up to 50% lower than control cells (Figure 4B). To determine if the effect on dTTP pools was a transient response to Fhit knockdown, we used A549 lung carcinoma cells that were engineered for stable silencing of Fhit expression. dNTPs were extracted from these cells after 7–9 weeks of stable Fhit-knockdown, and the dTTP pool was still significantly reduced relative to control cells (Figure 4C). While a 25–50% reduction in dTTP levels seems a modest change, it may suffice to hinder replication fork movement and cause DNA breaks, especially at loci sensitive to mild replication stress such as fragile sites. For example, low doses of hydroxyurea (0.1 mM) moderately reduce dNTP levels (by 20–40%) yet fully block DNA replication [28]. Moreover, treatment with 0.05 mM hydroxyurea significantly decreased replication fork movement and caused an increase in DNA breaks (Figure S3A–S3C). To determine if imbalance in the dTTP pool caused the DNA breaks in Fhit-deficient HEK293 cells, we supplemented Fhit-silenced cells with thymidine for 48 h, providing fresh thymidine every 24 h. Thymidine supplementation for 48 h resulted in an increase in dTTP pools in both control and Fhit-silenced HEK293 cells; thus, supplementation is sufficient to restore dTTP pools in Fhit-deficient cells (Figure S4). Measurement of comet tail moments showed that thymidine supplementation fully prevented the DNA breaks caused by Fhit knockdown (Figure 4D). Thymidine supplementation also corrected the DNA replication defects in Fhit-deficient cells, restoring fork velocity and improving sister fork symmetry (Figure 4E and 4F). We conclude that the supply of dTTP in the Fhit-deficient condition was inadequate to support efficient DNA replication.

**Figure 5:**
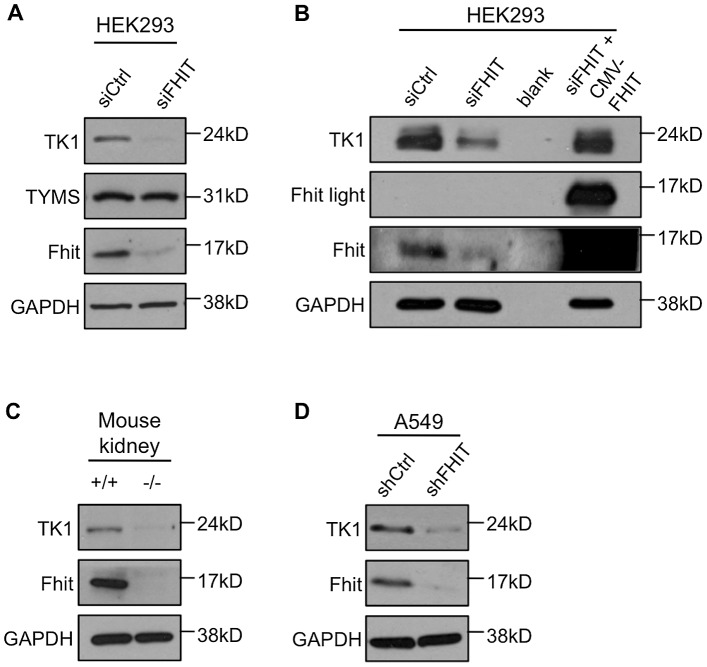
Fhit activates TK1 expression. (A) Western blot analysis of TK1 and TYMS expression in siRNA transfected HEK293 cells. Western blots were performed on 5 independent experiments, and a representative blot is shown. (B) Western blot analysis of TK1 expression in siRNA transfected HEK293 cells with or with exogenous Fhit overexpression. pRcCMV expression plasmid carrying *FHIT* cDNA was co-transfected with *FHIT* siRNAs to achieve exogenous Fhit overexpression. A representative blot is shown. (C) Western blot analysis of TK1 expression in *Fhit^+/+^* and *Fhit^−/−^* mouse kidney epithelial cells. A representative blot is shown. (D) Western blot analysis of TK1 expression in A549 cells with Fhit stably knocked down for 7–9 weeks. A representative blot is shown.

dTTP is synthesized by 2 pathways, the *de novo* pathway *via* Thymidylate synthase, TYMS; and the scavenger pathway *via* Thymidine kinase 1, TK1 [29]. Because of the observed effect on dTTP pools, we evaluated expression of TK1 and TYMS enzymes following Fhit knockdown. Expression of TYMS was unaffected by Fhit-silencing; in contrast, TK1 expression was severely depleted in HEK293 cells transfected with *FHIT* siRNAs (Figure 5A). Exogenous overexpression of Fhit in Fhit-deficient cells restored TK1 expression to normal levels (Figure 5B). *Fhit^−/−^* mouse kidney cells also expressed trace levels of TK1 compared to *Fhit^+/+^* cells (Figure 5C), and stable Fhit knockdown in A549 lung cancer cells caused TK1 down-modulation for at least 9 weeks (Figure 5D). Thus, Fhit modulation of TK1 expression is a general phenomenon, necessary for producing sufficient dTTP for DNA synthesis.

### The genome instability induced by Fhit-deficiency does not activate the S/G2 checkpoint

**Figure 6:**
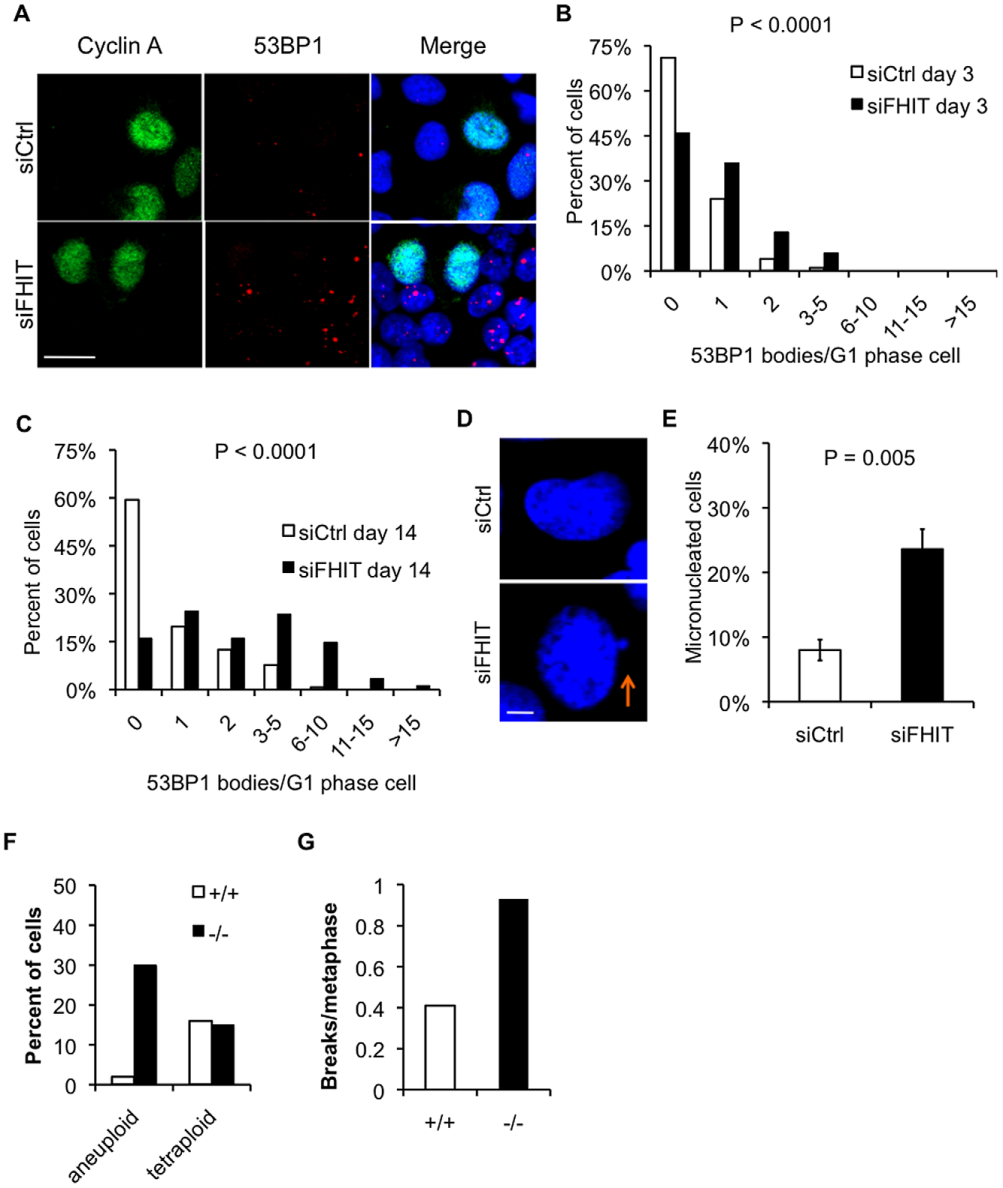
Loss of Fhit causes replication stress-induced chromosomal instability. (A) Cyclin A and 53BP1 immunofluorescence after Fhit knockdown in HEK293 cells. Representative images are shown; bars, 20 µm. (B and C) Histograms of 53BP1 nuclear bodies/G1 phase cell 3 days following siRNA transfection (B) or 14 days after siRNA transfection with fresh siRNAs transfected every 4 days (C). G1 phase cells were defined as cells negative for Cyclin A staining. Mann-Whitney rank sum test was used to determine P-values. (D) Representative images of DAPI-stained nuclei in siCtrl or siFHIT cells. Arrow marks a micronucleus; bars, 5 µm. (E) Quantification of micronucleated cells 3 days after siRNA transfections. Bar graphs represent the means, and error bars mark the standard deviations. P-value determined using a 2-sided T-test. (F) Percentage of aneuploid or tetraploid kidney cells established from *Fhit^+/+^* or *Fhit^−/−^* mouse kidney epithelial cells. Cells were sub-cultured 8 times and metaphase chromosomes were prepared and counted (n = 37 for *Fhit^+/+^*; n = 40 for *Fhit^−/−^* metaphases). (G) Quantification of the number of breaks/metaphase for the mouse kidney cells described in (F).

DSBs are the most deleterious DNA lesions as they are difficult to repair correctly and often lead to complex genomic alterations, including large deletions, duplications, and translocations [20]. Non-random mutation clusters can also arise during repair of DSBs [30], [31]. Cells minimize replication stress-induced DSB formation by activating the S phase replication checkpoint to block cell cycle progression and coordinate replication fork stabilization and restart [21]. Central to the S phase checkpoint are the kinases ATR and Chk1. ATR localizes to stalled forks and phosphorylates multiple targets, including Chk1. Phosphorylated Chk1 is then activated to phosphorylate its targets, setting off a cascade of events to enforce the S phase checkpoint [21]. We investigated the checkpoint response to Fhit-silencing in HEK293 cells, as these cells exhibit replication defects and spontaneous DSBs, first by immunoblot of lysates of HEK293 cells transfected with *FHIT* siRNAs, to assess expression of phospho-Chk1 (Ser317). We did not detect increased expression of phospho-Chk1 following Fhit knockdown, suggesting that the S phase checkpoint was not activated (Figure S5A). We also assessed the cell cycle distribution by flow cytometric analysis of DNA content to determine if Fhit-deficient cells accumulated in S or G2 phase. Consistent with the phospho-Chk1 western blot data, Fhit knockdown did not cause accumulation of cells in S or G2 phase (Figure S5B and S5C). While surprising, the results are not without precedent. For example, it is known that cells can traverse mitosis with under-replicated DNA due to replication stress and subsequently form DNA lesions marked by 53BP1 nuclear bodies in daughter G1 cells [32], [33]. Thus, we determined if Fhit-silenced cells complete cell division despite endogenous replication stress and DNA damage, by assessing the incidence of 53BP1 nuclear bodies in G1 cells by immunofluorescence staining of 53BP1 and Cyclin A. Within 3 days, Fhit knockdown in HEK293 cells led to a significant increase in the number of 53BP1 bodies in G1 phase cells, defined as the Cyclin A-negative cells (Figure 6A and 6B). Prolonged knockdown of Fhit in HEK293 cells, by transfecting fresh *FHIT* siRNAs every 4 days for 2 weeks, led to an even greater incidence of 53BP1 foci per G1 phase cell (Figure 6C). The results suggest that Fhit-deficient cells continue to proliferate and accumulate replication stress-induced DNA alterations.

In addition to the 53BP1-marked lesions, cells can acquire other replication stress-induced chromosomal aberrations during mitosis. For example, replication stress can cause micronucleus formation, due to failed segregation of chromosome fragments broken at fragile sites during mitosis or due to nondisjunction of chromosomes with incompletely replicated loci or unresolved replication intermediates [34], [35], events that result in large deletions or aneuploidy, respectively. In Fhit-silenced HEK293 cells, we observed an ∼3-fold increase in the percent of micronucleated cells relative to control cells (Figure 6D and 6E), confirming that replication stress caused chromosomal alterations. To measure aneuploidy incidence, we analyzed metaphase spreads from normal kidney cells established from *Fhit* ^+/+^ and *Fhit* ^−/−^ mice. At passage 8, nearly 30% of *Fhit* ^−/−^ cells were aneuploid, compared to fewer than 5% of the *Fhit* ^+/+^ cells (Figure 6F). In addition, *Fhit* ^−/−^ cells exhibited a 2-fold increase in the number of chromosome breaks/metaphase (Figure 6G). Taken together, the results show that Fhit loss–induced replication stress causes DNA lesions and chromosomal abnormalities following cell division in the absence of DNA damage checkpoint activation.

### Genomic alterations caused by Fhit loss expedite cell immortalization

**Figure 7:**
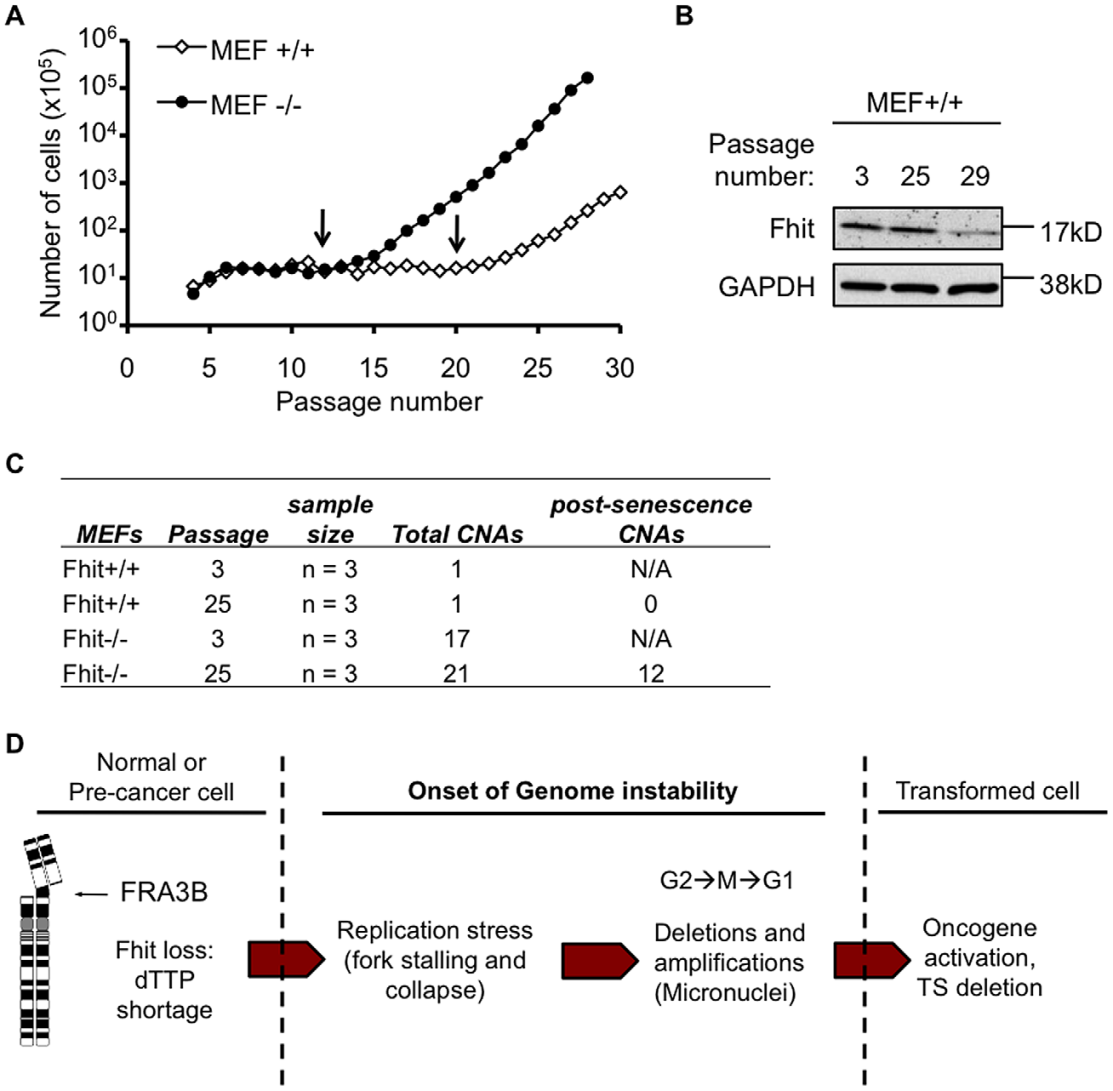
Genomic instability in Fhit-deficient cells correlates with onset of rapid proliferation and immortalization. (A) Analysis of *Fhit* ^+/+^ and *Fhit* ^−/−^ 3T3 MEF cell lines (n = 3, cell lines established from 3 embryos for each mouse strain). Arrows mark the passage numbers when MEFs became immortalized. (B) Western blot of *Fhit* ^+/+^ MEFs for Fhit and GAPDH expression. Immunoblots were performed on lysates obtained at the indicated passage number. (C) Summary of copy number aberrations (CNAs) in pre- and post-senescence MEFs from *Fhit* ^+/+^ and *Fhit* ^−/−^ mice. (D) The Fhit loss–induced genome instability model. Deletions in *FHIT* alleles occur due to FRA3B fragility causing loss of Fhit protein expression. Fhit loss causes dTTP pool insufficiency triggering replication stress, followed by stress-induced chromosomal instability. Chromosomal instability increases the likelihood of activating mutations in oncogenes and/or inactivating mutations in tumor suppressors, which are then selected for, facilitating cell transformation.

Because *FHIT* is an early target of allelic deletion in preneoplasia [4], [5], [36] and loss of Fhit protein expression induces replication stress, micronucleation and aneuploidy, we determined if Fhit-deficiency contributes to the onset of genomic instability in cells undergoing immortalization *in vitro*. Mouse embryo fibroblasts (MEFs) were established from *Fhit* ^+/+^ and *Fhit* ^−/−^ embryos (3 embryos per mouse strain) and were immortalized using the 3T3 protocol. *Fhit* ^−/−^ MEFs became immortalized and exhibited rapid growth at earlier tissue subcultures (passage 12, 14, and 16) compared to matching *Fhit^+/+^* MEFs (passage 14, 20, and 20) (Figure 7A). Fhit expression in *Fhit^+/+^* MEFs decreased as cells became immortalized (Figure 7B), and for the *Fhit* ^+/+^ MEF cell line showing rapid growth and immortalization by passage 14, a corresponding early loss of Fhit expression occurred. Therefore, loss of Fhit expression may be selected for as an essential step in the process of immortalization.

We then assessed somatic copy number aberrations, defined as DNA amplifications or deletions spanning more than 10 kb in size, using genomic DNA isolated from *Fhit* ^+/+^ and *Fhit* ^−/−^ MEFs grown in culture, pre- and post-senescence (at subcultures 3 and 25). Multiple copy number aberrations were detected in the pre- and post-senescent *Fhit* ^−/−^ MEFs, whereas only one was observed in one *Fhit^+/+^* MEF line (Figure 7C and Table S1). Somatic aberrations in the *Fhit* ^−/−^ MEFs occurred at 8 different genomic loci, 6 of which corresponded with fragile sites previously mapped in mouse fibroblasts [37] or lymphocytes [38] (Table S2), which implies that the genomic changes were caused by replication stress, and is consistent with reports that replication stress induces copy number changes [39], [40]. Across the 3 *Fhit* ^−/−^ MEF lines analyzed, 12 somatic aberrations occurred only in post-senescent cells, suggesting clonal expansion of cells harboring these genomic changes. Notably, 2 of the 3 *Fhit* ^−/−^ MEF cell lines acquired allelic gains within chromosome band 10D2, encompassing the murine *Mdm2* gene, an oncogene involved in cell transformation, and *Mdm2* gene amplification correlated with ∼4-fold increase in Mdm2 mRNA expression. These amplifications were likely selected for during immortalization, as they were present only in the cells that had escaped senescence.

Copy number aberrations were also observed in genomic DNA from *Fhit^−/−^* weanling tail tissue (Table S3). Most *Fhit* ^−/−^ tail DNA aberrations did not overlap with those observed in MEF cultures, suggesting that genomic instability is prevalent in *Fhit* ^−/−^ tissue early during development and copy number aberrations are selected during *in vitro* growth, depending on environment (eg, pre-senescent, senescent and post-senescent cultures). Genome instability has previously been observed in p53- and Gadd45a-deficient mice, where aneuploidy is detected in primary cells from multiple tissues [41]–[43]. Unlike the chromosomal instability in p53- and Gadd45a-deficient mouse cells, which exhibit aneuploidy due to centrosome amplification and mitotic errors, Fhit-deficient mice exhibit signs mostly of replication stress-induced DNA deletions and gains. Based on these observations and the fact that the genomic alterations begin in the knockout mouse tissue, we propose that the deletions within *FHIT* loci observed in preneoplastic human tissues *in vivo* initiate genomic instability and accelerate the neoplastic process.

## Discussion

### Model for Fhit loss-induced genome instability

This study has shown that loss of Fhit expression promotes the development of genomic instability. Fhit protein signals TK1 expression, and TK1 catalyzes the conversion of thymidine to dTMP as part of the scavenger pathway. This reaction contributes to dTTP pool production during S phase to support DNA synthesis. Consequently, loss of Fhit expression indirectly impairs replication fork progression, leading to fork stalling and DNA double-strand breaks. In Fhit-deficient cells, replication defects fail to activate the S or G2 checkpoints, and as cells complete mitosis, chromosomal alterations occur and are propagated to daughter cells. This process continues with each cell division cycle, and chromosomal instability inevitably arises. Upon acquisition of oncogenic mutations, selective pressures expedite cell transformation (see proposed model in Figure 7D).

Several alternative models have been proposed as common mechanisms for the origin of genome instability, including oxidative stress, telomere erosion, impaired DNA repair, and chromosome segregation errors; however, these forms likely do not contribute to the initiation of instability but rather to ongoing instability as they are seen in more advanced lesions [44]. The prevailing hypothesis for the origin of genome instability in preneoplastic cells is that defects in DNA replication result in DNA breaks and when incorrectly repaired, they produce chromosomal changes [20]. Thus, it is important to define the molecular source of replication stress that initiates genome instability. Oncogene activation can cause replication stress, chromosomal instability and promote tumorigenesis, and has been proposed as a mechanistic basis of genome instability [2], [44]. However, oncogene-induced replication stress is probably not the initiating event. For example, oncogene activation is achieved through various mechanisms that involve chromosome alterations, including translocations that change expression of the oncogene, duplications that increase the oncogene copy number, point mutations within the oncogene that increase its activity, deletions of a negative regulator, or epigenetic changes that affect gene expression. Also, because many oncogenes that induce senescence require a second genetic “hit” to uncouple mitogenic signaling from the senescence barrier [45], it seems probable that some degree of genetic instability and heterogeneity must precede oncogene activation.

There are two important distinctions in the Fhit-loss model that make it a more likely mechanism for the origin of genome instability. First, because of the inherent fragility at the FRA3B locus, the *FHIT* gene has been called the “weakest link” in the genome [46], making it a first target for inactivation in cells undergoing transformation, and its deletion a strong candidate initiator of genomic instability. Indeed, alterations at the *FHIT*/FRA3B locus are occasionally detected in normal cells without exposure to known inducing agent. These alterations can be caused by normal metabolic processes or by exposure to thus far undefined environmental stresses. The second important feature of the Fhit loss-induced genomic instability model is that cells acquire replication stress-induced chromosomal alterations without DNA damage response activation, in contrast to observations in oncogene-activated cells, possibly because the replication defects caused by Fhit loss fall below the threshold level needed to fully activate the S and G2 checkpoints. Likewise, aphidicolin induces fragile site expression by slowing or stalling replication forks, yet fragile sites are routinely detected in metaphase chromosomes, indicating a failure of the S and G2 checkpoints to block mitotic entry despite the presence of damaged loci. Furthermore, studies have suggested that eukaryotic cells lack a checkpoint surveillance mechanism to insure completion of DNA replication before mitotic entry [47]. Thus, it is possible that DNA replication is incomplete in Fhit-deficient cells because of a shortage in dTTP pools, and as cells pass through mitosis, under-replicated chromosomes either break or fail to properly segregate. In theory, without DNA damage checkpoint activation, Fhit-deficient cells could continue to proliferate for years, and over time accumulate extensive genome alterations generating significant mutational diversity and cell heterogeneity, as is the case with *Fhit* ^−/−^ mice. Indeed, *Fhit* ^−/−^ mice develop normally and live long lives, making *Fhit* inactivation an ideal target to initiate genome instability without compromising fitness at the cellular and organism levels. Thus, Fhit loss would provide the “soil” for the emergence of preneoplastic clones under selective pressure. This is consistent with the recent finding that clonal somatic chromosome anomalies increase with age in the normal population [9], [10] and is consistent with the fact that cancer risk increases with age. It is also consistent with the enhanced susceptibility of *Fhit^−/−^* mice to development of spontaneous tumors and their highly enhanced susceptibility to carcinogen-induced tumors [48].

### Fhit and the supply of dTTP

Mechanistically, the replication stress in Fhit-deficient cells was caused by a decrease in dTTP pools: silencing Fhit expression led to a moderate decrease in dTTP, a reduction sustained in stably Fhit-silenced cells; thymidine supplementation rescued the replication defects and suppressed DSBs in Fhit-silenced cells. Notably, there is a class of chromosome fragile sites, the folate-sensitive fragile sites, that are unstable under conditions that cause thymidylate depletion, including culturing in medium deficient in thymidine or folate [49]. Thymidine supplementation rescues the fragility of these sites. These findings independently establish that an insufficient supply of dTTP can cause chromosome instability at specific loci, and that the scavenger pathway, *via* TK1 activity, is a required source of dTTP to support DNA synthesis. Interestingly, folate is an important nutrient that serves as a cofactor for dTTP synthesis *via* activation of TYMS. Studies have shown that folate-deficiency correlates with several types of cancer, linking dTTP availability and tumorigenesis [50]. This is consistent with our findings that loss of Fhit decreases dTTP availability and promotes tumorigenesis.

dNTP pool depletion has been shown to affect DNA replication, cause genome instability, and is likely involved in oncogenic transformation [6]. Whereas oncogenes cause a dramatic decrease in dNTP levels, Fhit loss causes only moderate dTTP reduction, adequate to negatively affect DNA synthesis without blocking cell cycle progression. It is also relevant that deficiency of BLM helicase, in the highly penetrant autosomal recessive cancer-causing syndrome, is associated with a strong cytidine deaminase defect, leading to pyrimidine pool imbalance, specifically a 50% increase in the dCTP pool and only a 17% decrease in dTTP levels [51]. In BLM-deficient cells, thymidine supplementation leads to reduction of sister chromatid exchange frequency and is sufficient for full restoration of replication fork velocity. Fhit loss causes a more significant reduction of dTTP than in BLM-syndrome cells, ranging from a 25–50% decrease. It may be puzzling that thymidine supplementation can restore the supply of dTTP in Fhit-deficient cells, since TK1, down-modulated by Fhit-deficiency, is needed to convert thymidine to dTTP. Clearly TK1 is not completely absent in Fhit-silenced cells, since BrdU, CldU and IdU are also phosphorylated by TK1 prior to being incorporated into newly synthesized DNA. It is possible that by providing a continuous supply of thymidine for 48 h, even with low TK1 expression, Fhit-deficient cells accumulate a sufficient supply of dTTP leading up to S phase to support DNA synthesis.

TK1 is regulated in a cell cycle-dependent manner, with minimal expression during most of G1 phase; as cells prepare to start S phase, TK1 expression is dramatically up-regulated to contribute to dTTP biosynthesis [29]. Control of TK1 expression occurs at the transcriptional and the translation level [52]–[54]. TK1 protein expression remains high throughout S and G2 phase to facilitate sufficient dTTP production and as cells near the end of mitosis the enzyme is rapidly degraded to prevent overproduction of dTTP [55]. Accordingly, positive regulation of TK1 by Fhit may either occur through activation of mRNA transcription, protein translation or by restricting TK1 protein degradation to mitosis. Given that Fhit-deficient cells have decreased TK1 expression, it is probable that Fhit-deficient cells are reliant on TYMS, the *de novo* pathway, for synthesis of dTTP. This finding has clinical relevance as many chemotherapeutic agents, including 5-fluorouracil, target TYMS activity. Fhit expression in cancer cells may therefore be a predictor of cancer cell resistance to 5-fluorouracil, and administration of 5-fluorouracil to patients with Fhit-negative cancers may improve survival.

### *FHIT* deletion as a cancer-driving mutation

When common fragile sites were discovered in the early ‘80 s, it was noted that many of them mapped to loci that are non-randomly altered in cancers [56]. Thus it was thought that cloning of fragile sites would lead to discovery of genes that contribute to cancer development through genomic alteration. However, after the first fragile site gene was cloned and characterized as a cancer suppressor [57], it was suggested that *FHIT* and other fragile genes are altered in cancers due to their exquisite stress sensitivity rather than to a selective advantage imparted by loss of expression of fragile gene products [58]. An interesting twist to the story of fragile sites and cancer has been provided by the demonstration that the location of fragile sites in lymphoblasts and fibroblasts is different and dependent on the tissue specific, epigenetic determination of positions of DNA replication origins [59]–[61]; such tissue specificity of chromosome locations of common fragile sites had actually been observed much earlier for rodent and human tissues [62], [63]. Thus it is possible that the *FHIT* locus is not very fragile in epithelial cells, from which most cancers with *FHIT* deletions derive. If the FRA3B/*FHIT* locus is not the most fragile region in epithelial cells, then the fact that loss of heterozygosity at the *FHIT* gene is the most frequent alteration in cancer cells would suggest that loss of Fhit expression was a selected event in clonally expanded cells.

Our findings strongly support the view that loss of Fhit provides a selective advantage in sporadic cancers, directly or indirectly, because Fhit-deficient cells, which are genomically unstable, have a greater likelihood of acquiring cancer-promoting mutations. The relevance of Fhit loss during the neoplastic process has been inferred from the >50% frequency of Fhit loss in epithelial cancers [46], and from its tumor suppressor activity. The demonstration that *Fhit* ^−/−^ MEFs rapidly become immortalized and begin to acquire oncogenic DNA copy number aberrations, provide direct evidence of a genome ‘caretaker’ function for Fhit that is lost early in tumorigenesis. The development of sebaceous gland tumors in Fhit-deficient mice [64], a condition analogous to the sebaceous tumors of Muir-Torre syndrome in mismatch repair-deficient mice and humans [65], [66], and the observation that there are two forms of sebaceous tumors in humans, one form exhibiting mismatch repair gene deficiency and one exhibiting Fhit-deficiency [67], [68], can now be understood as a classic illustration of the caretaker function of Fhit. We conclude that Fhit loss is a common underlying initiator of genome instability in preneoplasia and a driver of the transformation process.

## Materials and Methods

### Ethics statement

The experiments involving isolation of mouse tissues for DNA analysis and for establishment of cell lines were done according to a protocol approved by the Ohio State University Institutional Animal Care and Use Committee (IACUC).

### Cell lines and reagents

HEK293 cells and *Fhit* ^+/+^ and *Fhit* ^−/−^ mouse kidney cells from C57Bl/B6 background mice, were cultured in MEM with 10% FBS and 100 µg/ml gentamicin. Fhit-deficient H1299 lung carcinoma cells were previously transfected with an inducible *FHIT* cDNA and tightly regulated inducible clones were isolated, including the D1 clone; empty vector control clones, including E1 cells, were also isolated [69]. For experiments using E1 and D1 cells, Fhit expression was induced by addition of ponasterone A (ponA) to growth medium (MEM, 10% FBS, gentamicin, zeocin and geneticin) for 48 h. A549 lung carcinoma cells with integration of a lentivirus containing shCtrl or shFHIT shRNAs were cultured in DMEM with 10% FBS, 100 µg/ml gentamicin and 1 µg/ml puromycin. For certain experiments, 2 mM hydroxyurea (Sigma) or 10 µM thymidine (Sigma) was added to cells for times indicated in text.

### siRNA transfections

HEK293 cells (60–80% confluent) were transfected with siRNAs targeting human *FHIT* or a non-specific control siRNA (Santa Cruz Biotechnology) using the manufacturer's recommended protocol. For each 60 mm^2^ dish, 1 µg of siRNAs and 5 µl of Lipofectamine 2000 (Invitrogen) were diluted in Opti-MEM (Gibco) and incubated for 30 min. Cells were washed in Opti-MEM, overlaid with the siRNA/Lipofectamine solution and incubated overnight at 37°C. Verification of siRNA knockdown of Fhit expression was performed 48–96 h later by Western blot.

### Comet assays

Neutral comet assays were performed using the CometAssay kit (Trevigen) and recommended protocol. Images were acquired with a Zeiss Axioskop 40 fluorescent microscope mounted with an AxioCam HRc camera, and using an A-Plan 10×/0.25 objective lens. Images were converted to Bitmap files using Axiovision 3.1 software, and comet tail moments were scored using Comet Score 1.5 (TriTek, autocomet.com).

### Immunofluorescence

Cells were grown on 8-chamber slides (Lab-Tek II), fixed with 4% paraformaldehyde, permeabilized with ice-cold 70% ethanol and blocked in 1% BSA. Primary antisera, rabbit anti-γH2AX, 1∶200 (Cell Signaling Technologies); rabbit anti-53BP1, 1∶200 (Cell Signaling Technologies); rabbit anti-pATR, 1∶100 (Cell Signaling Technologies); mouse anti-Cyclin A, 1∶100 (Santa Cruz Biotechnology); mouse anti-BrdU, 1∶100 (Millipore), were added and cells incubated with antisera overnight at 4°C. Slides were washed 3×10 min in PBS, and secondary antisera (AlexaFluor 488 or 594 - conjugated donkey anti-rabbit IgG or anti-mouse IgG, 1∶500, Molecular Probes) were added and incubated for 1 h at room temperature. Slides were washed and coverslips mounted using Fluoro-Gel II – with Dapi (Electron Microscope Sciences). Images were acquired at room temperature with an Olympus FV1000 spectral confocal microscope, a UPLFLN 40XO objective lens, NA 1.30, and with Olympus FLOWVIEW acquisition software. Brightness and contrast were adjusted equally for all images using Adobe Photoshop, and images were analyzed using Image J software.

### Western blot analysis

Cells were lysed with RIPA buffer (Thermo Scientific) supplemented with Halt Protease Cocktail Inhibitors (Thermo Scientific). Proteins were separated by SDS gel electrophoresis, transferred to nylon membranes and immunoblotted with antisera against human Fhit [70], GAPDH (Calbiochem), human TK1 (AbD serotec), mouse TK1 (Santa Cruz Biotechnology), or phospho-Chk1 (Ser 317) (Cell Signaling).

### Flow cytometry

siRNA transfected HEK293 cells were prepared for flow cytometric analysis of DNA content 72 h after transfection. Cells were harvested, fixed in ice-cold 70% ethanol at 4°C overnight. Cells were stained with propidium iodide solution (0.1 mg/ml propidium iodide, 0.1% Triton X-100, 0.2 µg/ml DNase-free RNase A) for 30 min and analyzed using a BD FACS Calibur.

### DNA fiber analysis

Cells were pulsed with 25 µM CldU for 30 min, washed, and pulsed with 250 µM IdU for 30 min. DNA fibers were prepared as described [24]. Cells were resuspended in PBS at 10^6^ cells/ml, 2 µl were spotted on glass sides and lysed with 5 µl of lysis buffer (0.5% SDS, 200 mM Tris-HCl, pH 7.4, 50 mM EDTA) for 10 min. Slides were tilted 15° to stretch DNA fibers by gravitational flow. Fibers were fixed with methanol/acetic acid (3∶1), denatured with 2.5 N HCl for 1 h, and blocked with 1% BSA. Rat anti-BrdU (1∶50, AbD Serotec) was used to detect CldU, and mouse anti-BrdU (1∶20, Becton Dickinson) to detect IdU. Primary antibodies were fixed with 4% paraformaldehyde and detected with AlexaFluor 594 – conjugated donkey anti-rat IgG (1∶250, Molecular Probes) and AlexaFluor 488 – conjugated donkey anti-mouse IgG (1∶250, Molecular Probes). Coverslips were mounted using Fluoro-Gel II – with Dapi (Electron Microscope Sciences). Images were acquired at room temperature using an Olympus FV1000 spectral confocal microscope and with a PLAPON 60XO objective lens, NA 1.42. Fiber lengths were measured using Olympus FLUOVIEW software, and velocities were estimated using a conversion factor of 1 µM = 2.59 kbp. Brightness and contrast were adjusted equally for all images using Adobe Photoshop.

### Preparation of metaphase spreads

Metaphase chromosomes were prepared from *Fhit* ^+/+^ or *Fhit* ^−/−^ mouse kidney cells at the 8^th^ subculture. Cells were treated with colcemid (0.1 µg/ml) for 1 h to block cells in mitosis. Cells were trypsinized, pelleted and resuspended in 0.075 M KCl hypotonic solution for 10 min at 37°C. Cells were fixed in methanol/acetic acid (3∶1), dropped on glass slides and allowed to air dry. Chromosomes were analyzed using a Zeiss Axioskop Widefield LM at 100× magnification.

### dNTP pool measurements

HEK293 cells transfected with si*FHIT* or siCtrl were cultured in 10 cm^2^ dishes for 72 h and dNTPs were extracted as previously described [71]. Exponentially growing cells were washed twice with ice-cold PBS, covered in ice-cold methanol and incubated at −20°C for 1–3 h. An additional plate of exponentially growing cells was used to calculate the total cell number per extract. Cell extracts were collected and incubated in boiling water for 3 min, and separated from cell debris by centrifugation at 17,000× g, for 10 min at 4°C. dNTP extracts were dried using a Speed vacuum. dNTP pools were assayed using the enzymatic method. For each dNTP to be measured an oligonucleotide template was used [72]. The same primer was used for all four assays. The reaction mixture was mixed with either the dNTP extracts or known standards used to construct a standard curve, and DNA polymerization was carried out at 37°C for 45 min. Reaction mixtures were spotted on DE81 chromatography paper, dried and washed in 5% Na_2_HPO_4_, followed by water, and finally in 95% ethanol. Samples were counted using a liquid scintillation counter. dNTP concentrations were determined by reference to the standard curve.

### 3T3 cell culture and analysis of cell growth kinetics

Mouse embryonic fibroblasts were isolated from individual 13-day embryos of *Fhit* ^+/+^ and *Fhit* ^−/−^ pregnant females and cultured in Dulbecco Modified Eagle's Medium with 10% fetal bovine serum and 100 µg/ml gentamicin. Primary MEFs were subcultured by trypsinizing and replating 3×10^5^ cells per 6 cm^2^ dish every three days (3T3 protocol). The time of immortalization was defined retrospectively as the first tissue culture passage after which the cell population increased consistently with each subculture.

### Copy number variation analysis

Genomic DNA was isolated from *Fhit* ^+/+^ and *Fhit* ^−/−^ MEFs at tissue culture passages 3 and 25 (n = 3 lines, 1 from each of 3 embryos, for each pair at each passage) using DNeasy Blood and Tissue (Qiagen). Genomic DNA was also isolated from *Fhit* ^+/+^ and *Fhit* ^−/−^ weanling tail DNA. Genomic DNA samples were analyzed for copy number aberrations at Jackson Labs using the Affymetrix Mouse Diversity Genotype Array. *Fhit* ^+/+^ tail DNA served as reference DNA.

### Statistical analysis

For all boxplots, bottom and top of the box correspond to the 25^th^ and 75^th^ percentiles, respectively, and whiskers represent data points within 1.5×IQR (interquartile range). The gray line extending through the boxplot indicates the mean value, and the black line contained within the boxplot represents the median value. Two-sided T-tests were used to determine significance for data with a normal distribution and equal variances. Nonparametric data was analyzed using the Mann-Whitney rank sum test for single comparisons or using the Kruskal-Wallis test for multiple comparisons. Groups with P-values less than an alpha of 0.05 were considered significantly different.

## Supporting Information

Figure S1 Western blot analysis of Fhit expression in HEK293 and H1299 cells. (A) siRNA knockdown of Fhit protein expression 48 h after transfection. (B) Ponasterone A – induction of Fhit expression in H1299 D1 cells. Cells were treated with ponasterone A, final concentration of 5 µM, and incubated for 3 days. D1 clones contain the ponasterone A – inducible *FHIT* cDNA expression plasmids. E1 clones contain the empty vector controls. ponA = ponasterone A, 5 µM.
(TIF)

Figure S2 DNA breaks occur during S phase in Fhit deficient cells. (A) Immunofluorescence of Cyclin A and γH2AX in H1299 E1 and D1 cells 48 h after ponA addition. Representative images are shown. (B) Quantification of γH2AX-only or γH2AX and Cyclin A-positive E1 and D1 cells. (C) Immunofluorescence of BrdU-incorporation and γH2AX in E1 and D1 cells 48 h after ponA addition. BrdU was added for 15 min before fixing. Representative images are shown. (D) Quantification of γH2AX-only or γH2AX and BrdU-positive E1 and D1 cells. (E) Immunofluorescence of γH2AX in E1 cells 6 h after treatment with roscovitine or mock treatment. (F) Quantification of γH2AX-positive E1 and D1 cells.
(TIF)

Figure S3 Low hydroxyurea concentration reduces fork speed and causes DNA breaks. (A) Illustration of experimental design. PonA-treated H1299 E1 and D1 cells were cultured in the presence of hydroxyurea (50 uM) for 48 h, then sequentially pulsed with CldU and IdU. (B) DNA fiber analysis of fork velocity in H1299 E1 and D1 cells cultured as in (A). (C) Comet assay analysis of DNA breaks in H1299 E1 and D1 cells cultured as in (A).
(TIF)

Figure S4 Thymidine supplementation restores dTTP pools in Fhit-silenced cells. dNTP pools in siRNA transfected HEK293 cells supplemented daily with thymidine 10 µM for 48 h. Bar graphs illustrate the means of 1 experiment performed in quadruplicate. Error bars show the standard deviations. dT = thymidine.
(TIF)

Figure S5 Fhit knockdown in HEK293 does not activate the DNA damage checkpoint. (A) Western blots of phospho-Chk1 (Ser317), Fhit, and GAPDH expression in HEK293 cells following siRNA transfections. (B) Cell cycle distributions of HEK293 cells 4 days after siRNA transfections. (C) Flow cytometric analysis of DNA content in HEK293 cells 4 days after siRNA transfections.
(TIF)

Table S1 Complete list of copy number aberrations in Fhit+/+ and Fhit−/− MEFs. List of copy number aberrations (gains and losses) detected in genomes of MEF cell lines from Fhit+/+ or Fhit−/− mice at passage 3 or 25. MEFs were established from 3 different embryos for each genotype. NA, not applicable as no CNAs were detected in the DNA of these MEF cell lines.
(DOCX)

Table S2 CNAs occur predominantly at fragile loci. List of the loci where CNAs were detected in Fhit−/− MEFs. Many of these loci were previously shown to be fragile in MEFs or in mouse lymphocytes. Medium expression and high expression refer to the frequency of breaks detected at a given locus following mild replication stress induced by aphidicolin. Loci with high expression develop breaks at a high frequency; loci with medium expression develop breaks but at a lower frequency than the high expression loci; and non-fragile loci rarely develop breaks.
(DOCX)

Table S3 Copy number aberrations in Fhit−/− tail tissue. List of copy number aberrations (all losses) in DNA isolated from mouse tail tissue. Four of the deleted loci were also observed in DNA of Fhit−/− MEF cell lines, whereas the remaining 12 deleted loci were unique to the tail tissue.
(DOCX)
