# Supplementary figures and images for: Initiation of Genome Instability and Preneoplastic Processes through Loss of Fhit Expression
Source: PLoS Genet. 2012 Nov 29;8(11):e1003077. doi: 10.1371/journal.pgen.1003077 (PMC3510054; doi:10.1371/journal.pgen.1003077)

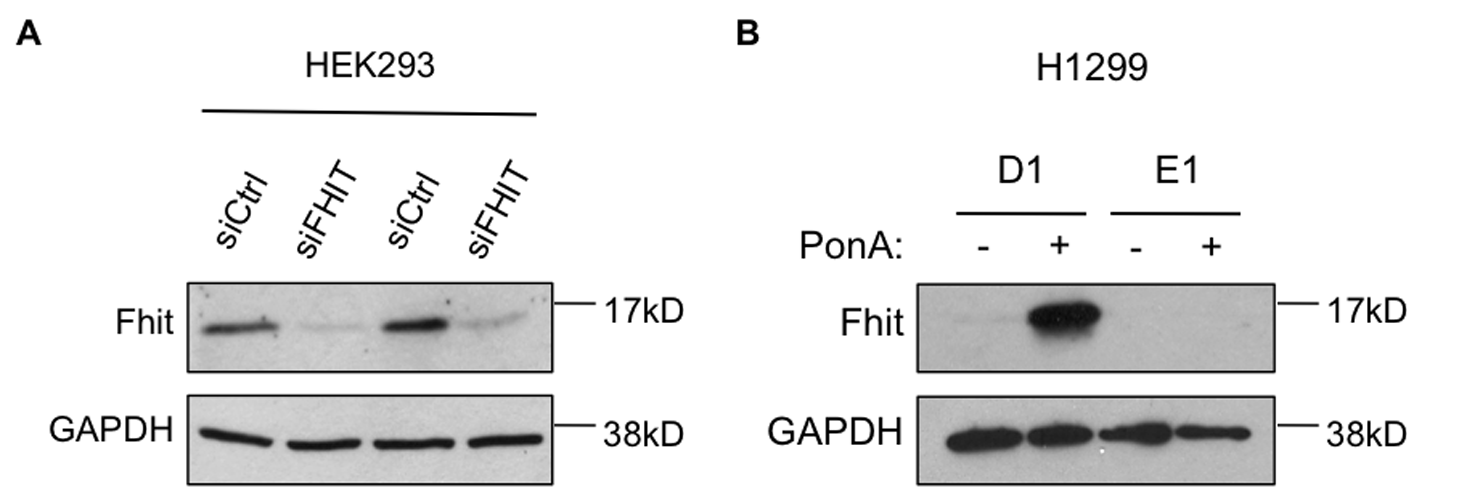

Supplement: Figure S1 — Western blot analysis of Fhit expression in HEK293 and H1299 cells. (A) siRNA knockdown of Fhit protein expression 48 h after transfection. (B) Ponasterone A – induction of Fhit expression in H1299 D1 cells. Cells were treated with ponasterone A, final concentration of 5 µM, and incubated for 3 days. D1 clones contain the ponasterone A – inducible FHIT cDNA expression plasmids. E1 clones contain the empty vector controls. ponA = ponasterone A, 5 µM. (TIF) [file pgen.1003077.s001.tif]

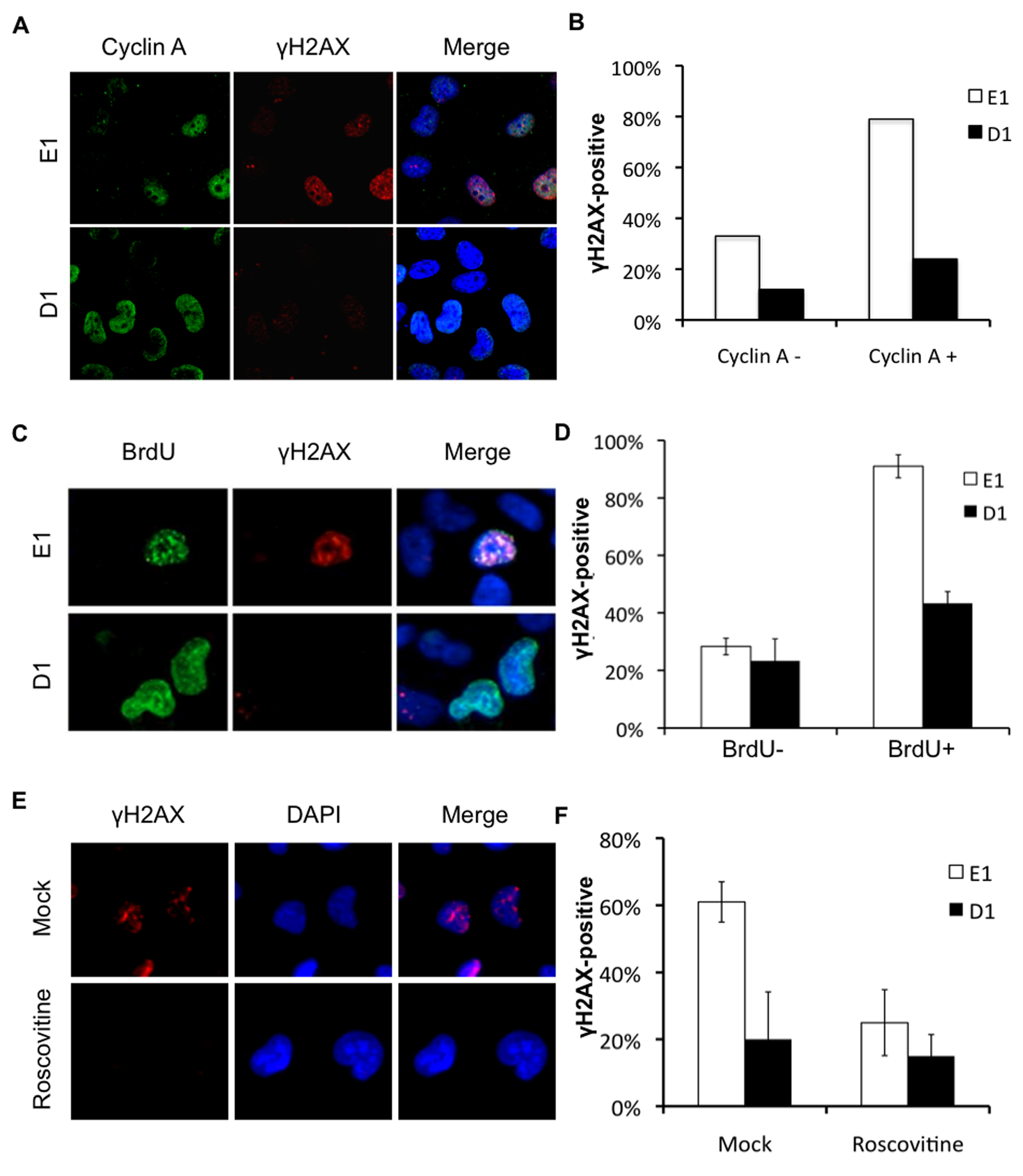

Supplement: Figure S2 — DNA breaks occur during S phase in Fhit deficient cells. (A) Immunofluorescence of Cyclin A and γH2AX in H1299 E1 and D1 cells 48 h after ponA addition. Representative images are shown. (B) Quantification of γH2AX-only or γH2AX and Cyclin A-positive E1 and D1 cells. (C) Immunofluorescence of BrdU-incorporation and γH2AX in E1 and D1 cells 48 h after ponA addition. BrdU was added for 15 min before fixing. Representative images are shown. (D) Quantification of γH2AX-only or γH2AX and BrdU-positive E1 and D1 cells. (E) Immunofluorescence of γH2AX in E1 cells 6 h after treatment with roscovitine or mock treatment. (F) Quantification of γH2AX-positive E1 and D1 cells. (TIF) [file pgen.1003077.s002.tif]

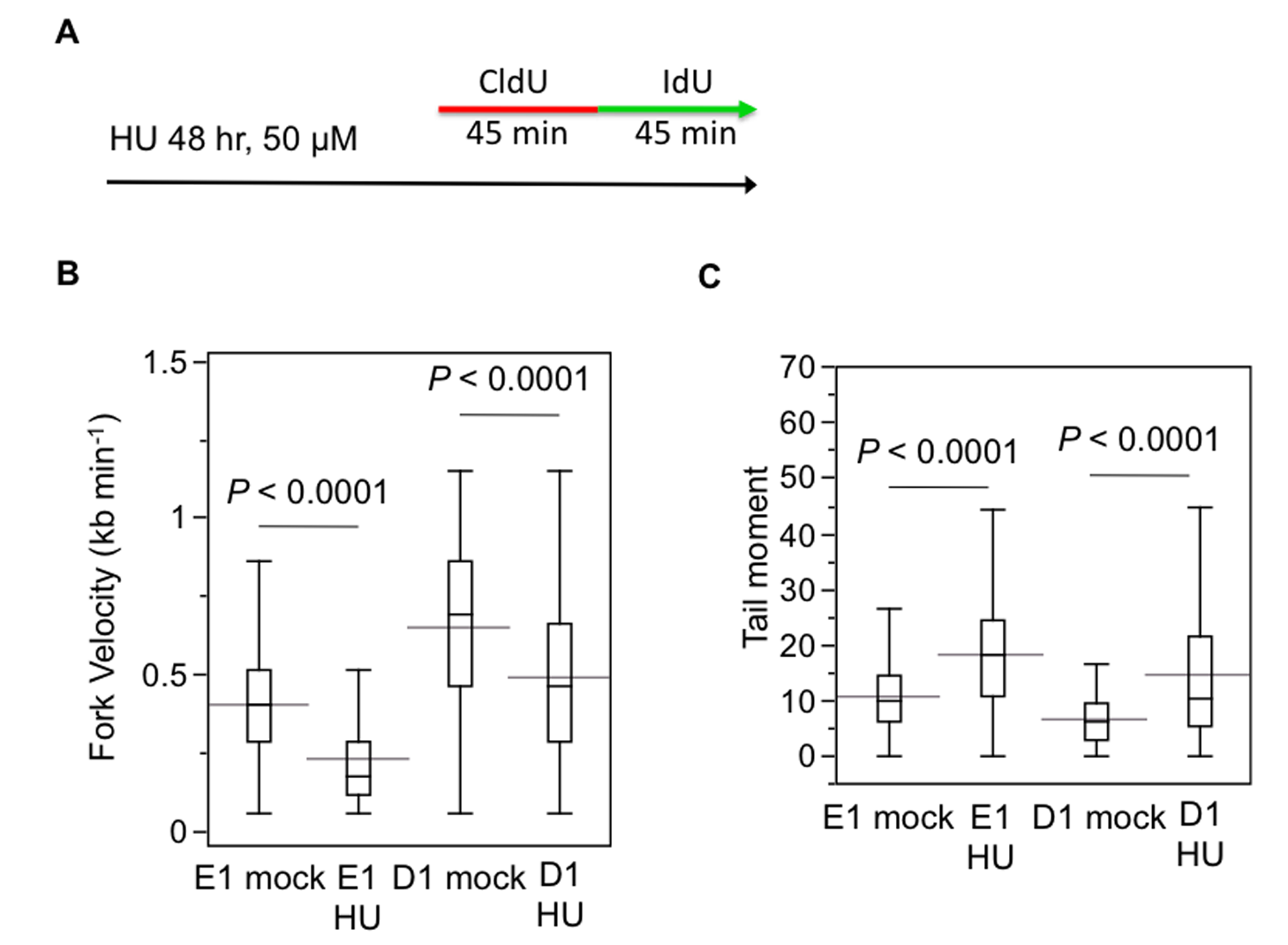

Supplement: Figure S3 — Low hydroxyurea concentration reduces fork speed and causes DNA breaks. (A) Illustration of experimental design. PonA-treated H1299 E1 and D1 cells were cultured in the presence of hydroxyurea (50 uM) for 48 h, then sequentially pulsed with CldU and IdU. (B) DNA fiber analysis of fork velocity in H1299 E1 and D1 cells cultured as in (A). (C) Comet assay analysis of DNA breaks in H1299 E1 and D1 cells cultured as in (A). (TIF) [file pgen.1003077.s003.tif]

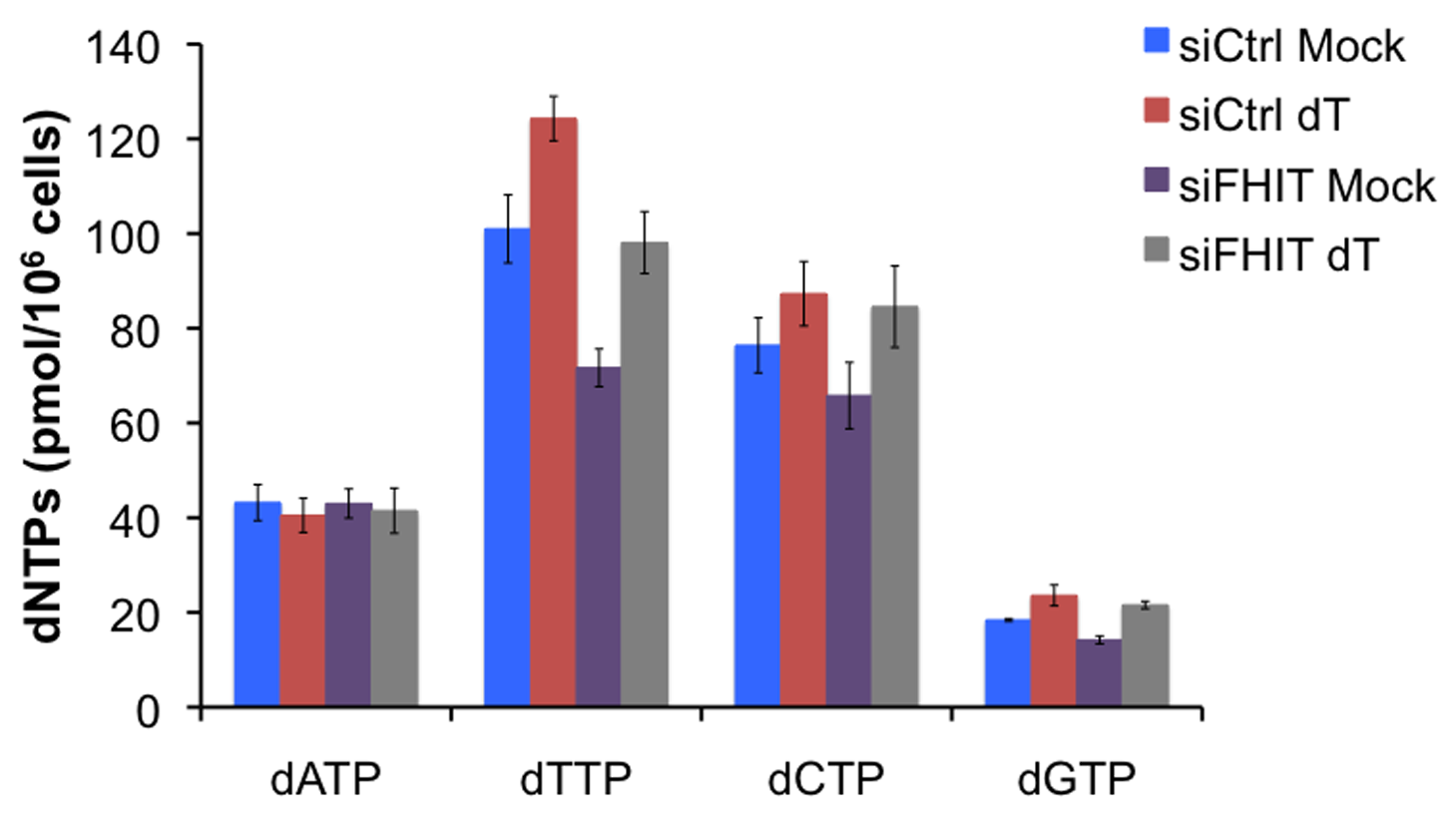

Supplement: Figure S4 — Thymidine supplementation restores dTTP pools in Fhit-silenced cells. dNTP pools in siRNA transfected HEK293 cells supplemented daily with thymidine 10 µM for 48 h. Bar graphs illustrate the means of 1 experiment performed in quadruplicate. Error bars show the standard deviations. dT = thymidine. (TIF) [file pgen.1003077.s004.tif]

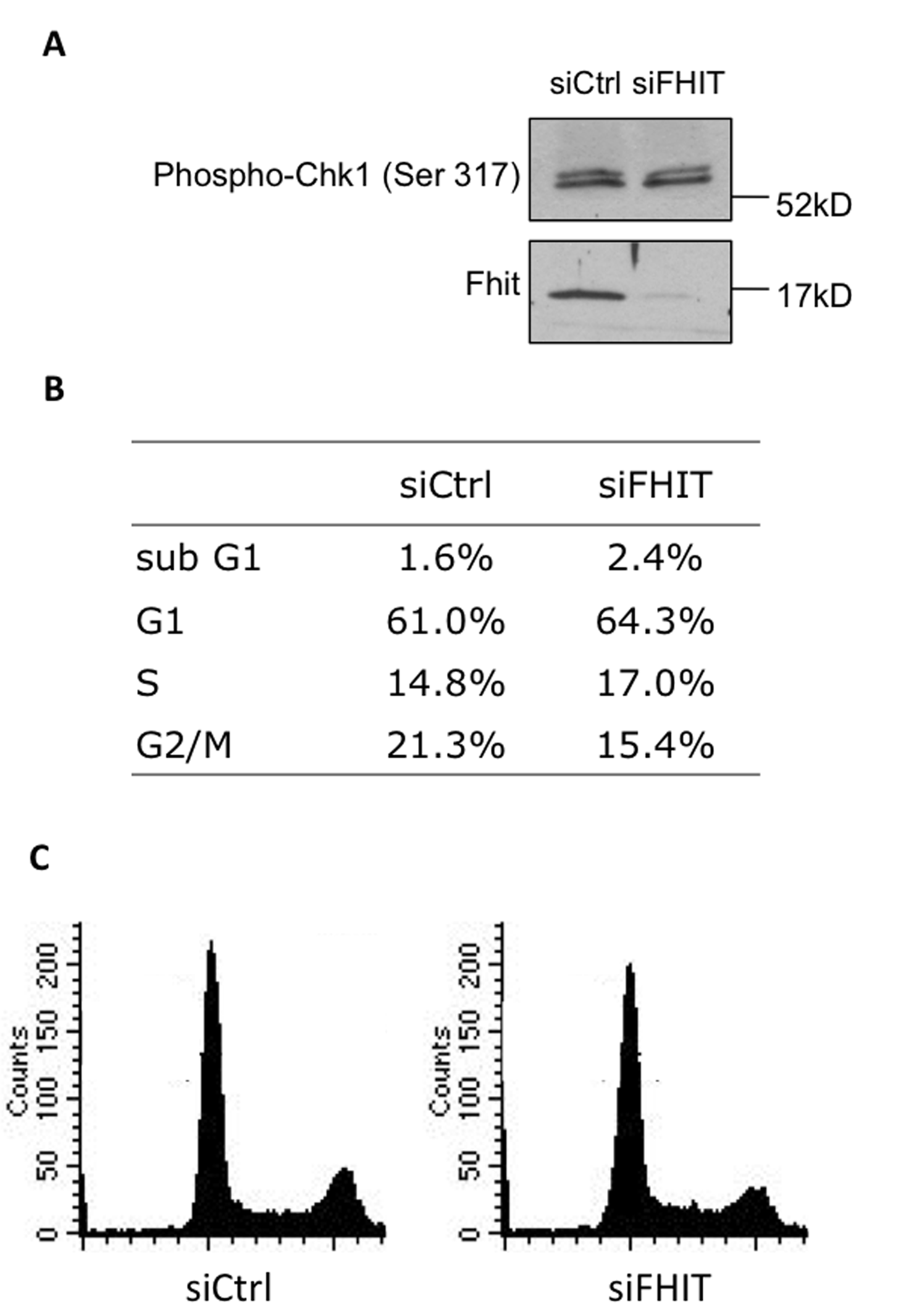

Supplement: Figure S5 — Fhit knockdown in HEK293 does not activate the DNA damage checkpoint. (A) Western blots of phospho-Chk1 (Ser317), Fhit, and GAPDH expression in HEK293 cells following siRNA transfections. (B) Cell cycle distributions of HEK293 cells 4 days after siRNA transfections. (C) Flow cytometric analysis of DNA content in HEK293 cells 4 days after siRNA transfections. (TIF) [file pgen.1003077.s005.tif]
