# Supplementary material for: Initiation of Genome Instability and Preneoplastic Processes through Loss of Fhit Expression
Source: PLoS Genet. 2012 Nov 29;8(11):e1003077. doi: 10.1371/journal.pgen.1003077 (PMC3510054; doi:10.1371/journal.pgen.1003077)
Supplement: Table S1 — Complete list of copy number aberrations in Fhit+/+ and Fhit−/− MEFs. List of copy number aberrations (gains and losses) detected in genomes of MEF cell lines from Fhit+/+ or Fhit−/− mice at passage 3 or 25. MEFs were established from 3 different embryos for each genotype. NA, not applicable as no CNAs were detected in the DNA of these MEF cell lines. (DOCX) [file pgen.1003077.s006.docx]

**Table S1.** Complete list of copy number aberrations in Fhit+/+ and Fhit-/- MEFs.

| ***MEFs*** | ***Chromosome*** | ***Status*** | ***# of Genes*** | ***Genes*** | ***Span*** | ***Size*** |
| --- | --- | --- | --- | --- | --- | --- |
| +/+ mouse 1 passage 3 | NA | NA | NA | NA | NA | NA |
| +/+ mouse 1 passage 25 | NA | NA | NA | NA | NA | NA |
| +/+ mouse 2 passage 3 | NA | NA | NA | NA | NA | NA |
| +/+ mouse 2 passage 25 | NA | NA | NA | NA | NA | NA |
| +/+ mouse 3 passage 3 | 3A3 | loss | 1 | Nlgn1 | 25426121-25461978 | 35858 |
| +/+ mouse 3 passage 25 | 3A3 | loss | 1 | Nlgn1 | 25426121-25445541 | 19421 |
| -/- mouse 3 passage 3 | 3F2.3-3F3 | loss | 4 | Chia, Chi3l3, Chi3l4, Gm6522 | 105931179-106110585 | 179407 |
| -/- mouse 3 passage 3 | 13A1 | loss | 1 | Gm7446 | 13974389-13987563 | 13175 |
| -/- mouse 3 passage 3 | 16B3 | loss | 4 | 2010005H15Rik, Stfa1, Gm4758, BC117090 | 36257364-36321838 | 64475 |
| -/- mouse 3 passage 25 | 8C2-8C3 | loss | 2 | Scoc, Gm5910 | 85975086-86023239 | 48154 |
| -/- mouse 3 passage 25 | 10D2 | gain | 18 | Tbc1d15, Gm8942, Rab21, Gm10752, Tmem19, LOC100418112, Thap2, Zfc3h1, Lgr5, A930009a15Rik, Gm8960, Tspan8, 4933416C03Rik, Ptprr, LOZC100504392, Ptprb, 1700058G18Rik, Kenmb4, | 114646006-115910232 | 1264227 |
| -/- mouse 3 passage 25 | 10D2 | gain | 3 | LOC100504412, Cnot2, LOC100504423 | 115911553-116013720 | 102168 |
| -/- mouse 3 passage 25 | 10D2 | gain | 3 | LOC100504423, Gm8965, Gm239 | 116019245-116219080 | 199836 |
| -/- mouse 3 passage 25 | 10D2 | gain | 24 | Gm239, Rab3ip, 4933412E12Rik, D630029K05Rik, Gm5781, Best3, Gm10747, Lrrc10, Cct2, Frs2, Yeats4, 9530003J23Rik, Lyz2, Lyz1, Cpfs6, Gm9002, Kifc5c, Gm9004, Cpm, Mdm2, Slc35e3, Nup107, LOC100504499, Rap1b | 116233152-117408874 | 1175723 |
| -/- mouse 3 passage 25 | 13A1 | loss | 3 | Ero1lb, LOC100502964, Gpr137b-ps | 12699132-12720056 | 20925 |
| -/- mouse 3 passage 25 | 13A1 | loss | 1 | Gm7446 | 13974389-13987592 | 13204 |
| -/- mouse 3 passage 25 | 16B3 | loss | 4 | 2010005H15Rik, Stfa1, Gm4758, BC117090 | 36257364-36321838 | 64475 |
| -/- mouse 4 passage 3 | 3A3 | loss | 1 | Nlgn1 | 25426121-25445541 | 19421 |
| -/- mouse 4 passage 3 | 4E1 | loss | 8 | Gm13043, Gm13040, Gm13057, BC080695, Gm13080, LOC100044633, Gm13083, Gm13088 | 143092288-143250513 | 158226 |
| -/- mouse 4 passage 3 | 4E1 | loss | 2 | Gm13109, Gm13101 | 143540903-143555553 | 14651 |
| -/- mouse 4 passage 3 | 8C2-8C3 | loss | 2 | Scoc, Gm5910 | 85975086-86023239 | 48154 |
| -/- mouse 4 passage 3 | 13A1 | loss | 3 | Ero1lb, LOC100502964, Gpr137b-ps | 12699208-12720056 | 20849 |
| -/- mouse 4 passage 3 | 13A1 | loss | 1 | Gm7446 | 13974389-13987592 | 13204 |
| -/- mouse 4 passage 3 | 16B3 | loss | 4 | 2010005H15Rik, Stfa1, Gm4758, BC117090 | 36257364-36321838 | 64475 |
| -/- mouse 4 passage 25 | 3A3 | loss | 1 | Nlgn1 | 25426121-25445541 | 19421 |
| -/- mouse 4 passage 25 | 4E1 | loss | 2 | Gm13109, Gm13101 | 143540903-143555553 | 14651 |
| -/- mouse 4 passage 25 | 13A1 | loss | 3 | Ero1lb, LOC100502964, Gpr137b-ps | 12696661-12720056 | 23396 |
| -/- mouse 4 passage 25 | 13A1 | loss | 1 | Gm7446 | 13972917-13987592 | 14676 |
| -/- mouse 4 passage 25 | 16B3 | loss | 4 | 2010005H15Rik, Stfa1, Gm4758, BC117090 | 36257364-36321838 | 64475 |
| -/- mouse 5 passage 3 | 3A3 | loss | 1 | Nlgn1 | 25426121-25471172 | 45052 |
| -/- mouse 5 passage 3 | 3F2.3-3F3 | loss | 4 | Chia, Chi3l3, Chi3l4, Gm6522 | 105933348-106110585 | 177238 |
| -/- mouse 5 passage 3 | 4E1 | loss | 3 | LOC100044633, Gm13083, Gm13088 | 143201660-143250513 | 48854 |
| -/- mouse 5 passage 3 | 4E1 | loss | 2 | Gm13109, Gm13101 | 143540903-143555553 | 14651 |
| -/- mouse 5 passage 3 | 8C1 | loss | 1 | Ttc29 | 80806273-80834051 | 27779 |
| -/- mouse 5 passage 3 | 13A1 | loss | 1 | Gm7446 | 13974389-13987592 | 13204 |
| -/- mouse 5 passage 3 | 16B3 | loss | 4 | 2010005H15Rik, Stfa1, Gm4758, BC117090 | 36257364-36321838 | 64475 |
| -/- mouse 5 passage 25 | 3A3 | loss | 1 | Nlgn1 | 25426121-25445541 | 19421 |
| -/- mouse 5 passage 25 | 3F2.3-3F3 | loss | 4 | Chia, Chi3l3, Chi3l4, Gm6522 | 105933348-106110585 | 177238 |
| -/- mouse 5 passage 25 | 4E1 | loss | 2 | Gm13109, Gm13101 | 143540903-143555553 | 14651 |
| -/- mouse 5 passage 25 | 8C1 | loss | 1 | Ttc29 | 80806273-80821978 | 15706 |
| -/- mouse 5 passage 25 | 10D2 | gain | 40 | Best3, Gm10747, Lrrc10, Cct2, Frs2, Yeats4, 9530003J23Rik, Lyz2, Lyz1, Cpfs6, Gm9002, Kifc5c, Gm9004, Cpm, Mdm2, Slc35e3, Nup107, LOC100504499, Rap1b, Mdm1, Il22, Gm9585, Gm9029, Gm9030, Gm9035, Iltifb, Gm9044, Ifng, Dyrk2, LOC100417929, Cand1, LOC100418236, Grip1 | 116433036-119040590 | 2607555 |
| -/- mouse 5 passage 25 | 13A1 | loss | 3 | Ero1lb, LOC100502964, Gpr137b-ps | 12699208-12720056 | 20849 |
| -/- mouse 5 passage 25 | 13A1 | loss | 1 | Gm7446 | 13972917-13987563 | 14647 |
| -/- mouse 5 passage 25 | 16B3 | loss | 4 | 2010005H15Rik, Stfa1, Gm4758, BC117090 | 36257364-36321838 | 64475 |
