# Supplementary material for: Initiation of Genome Instability and Preneoplastic Processes through Loss of Fhit Expression
Source: PLoS Genet. 2012 Nov 29;8(11):e1003077. doi: 10.1371/journal.pgen.1003077 (PMC3510054; doi:10.1371/journal.pgen.1003077)
Supplement: Table S2 — CNAs occur predominantly at fragile loci. List of the loci where CNAs were detected in Fhit−/− MEFs. Many of these loci were previously shown to be fragile in MEFs or in mouse lymphocytes. Medium expression and high expression refer to the frequency of breaks detected at a given locus following mild replication stress induced by aphidicolin. Loci with high expression develop breaks at a high frequency; loci with medium expression develop breaks but at a lower frequency than the high expression loci; and non-fragile loci rarely develop breaks. (DOCX) [file pgen.1003077.s007.docx]

**Table S2.**  CNAs occur predominantly at fragile loci.

| ***CNA locus*** | ***MEF fragile site*** | ***Mouse lymphocyte fragile site*** |
| --- | --- | --- |
| **3A3** | **no** | **Yes (medium expression)** |
| **3F2.3-3F3** | **yes** | **Yes (medium expression)** |
| **4E1** | **no** | **no** |
| **8C1** | **yes** | **Yes (high expression)** |
| **8C2-8C3** | **yes** | **Yes (high expression)** |
| **10D2** | **yes** | **Yes (medium expression)** |
| **13A1** | **no** | **no** |
| **16B3** | **no** | **Yes (medium expression)** |
