# Supplementary material for: Initiation of Genome Instability and Preneoplastic Processes through Loss of Fhit Expression
Source: PLoS Genet. 2012 Nov 29;8(11):e1003077. doi: 10.1371/journal.pgen.1003077 (PMC3510054; doi:10.1371/journal.pgen.1003077)
Supplement: Table S3 — Copy number aberrations in Fhit−/− tail tissue. List of copy number aberrations (all losses) in DNA isolated from mouse tail tissue. Four of the deleted loci were also observed in DNA of Fhit−/− MEF cell lines, whereas the remaining 12 deleted loci were unique to the tail tissue. (DOCX) [file pgen.1003077.s008.docx]

**Table S3.** Copy number aberrations in Fhit-/- tail tissue.

| ***Genotype*** | ***Chromosome*** | ***Status*** | ***# of genes*** | ***Genes*** | ***Span*** | ***Size*** |
| --- | --- | --- | --- | --- | --- | --- |
| -/- tail | 2D | loss | 2 | Olfr141, Olfr1094 | 86646195-86670052 | 23858 |
| -/- tail | 3F2.3-3F3^a^ | loss | 4 | Chia, Chi3l3, Chi3l4, Gm6522 | 105933348-106110585 | 177238 |
| -/- tail | 4A4-4A5 | loss | 0 | N/A | 27102368-27307050 | 204683 |
| -/- tail | 4A4 | loss | 0 | N/A | 29717750-29825488 | 107739 |
| -/- tail | 6C2 | loss | 0 | N/A | 75342461-75464922 | 122462 |
| -/- tail | 8A4 | loss | 1 | Sgcz | 38773861-38831368 | 57508 |
| -/- tail | 8B1.3 | loss | 0 | N/A | 52224120-52273067 | 48948 |
| -/- tail | 8B1.3 | loss | 1 | LOC100416238 | 52879756-52943783 | 64028 |
| -/- tail | 8B1.3 | loss | 2 | Gm9892, Gm6463 | 53127775-53364507 | 236733 |
| -/- tail | 8B1.3-8B2 | loss | 0 | N/A | 55941523-56033681 | 92159 |
| -/- tail | 8C1^a^ | loss | 1 | Ttc29 | 80806273-80821978 | 15706 |
| -/- tail | 12B2 | loss | 0 | N/A | 44361695-44464705 | 103011 |
| -/- tail | 13A1^a^ | loss | 1 | Gm7446 | 13972917-13987592 | 14676 |
| -/- tail | 15B3.3-15C | loss | 0 | N/A | 49746931-49777457 | 30527 |
| -/- tail | 16B2 | loss | 0 | N/A | 27642302-27784650 | 142349 |
| -/- tail | 16B3^a^ | loss | 4 | 2010005H15Rik, Gm 13040, Gm13057, BC080695 | 36257364-36321838 | 64475 |
| -/- tail | 17D-17E1.1 | loss | 1 | LOC100417309 | 60730307-60831232 | 100926 |

**^a^**CNAs also observed in Fhit-/- MEFs.
